# A Three‐in‐One Hybrid Strategy for High‐Performance Semiconducting Polymers Processed from Anisole

**DOI:** 10.1002/advs.202401345

**Published:** 2024-04-22

**Authors:** Cheng Liu, Huanhuan Liang, Runze Xie, Quanfeng Zhou, Miao Qi, Chongqing Yang, Xiaodan Gu, Yunfei Wang, Guoxiang Zhang, Jinlun Li, Xiu Gong, Junwu Chen, Lianjie Zhang, Zesheng Zhang, Xiang Ge, Yuanyu Wang, Chen Yang, Yi Liu, Xuncheng Liu

**Affiliations:** ^1^ College of Materials and Metallurgy Guizhou University Guiyang 550025 P. R. China; ^2^ The Molecular Foundry Lawrence Berkeley National Laboratory One Cyclotron Road Berkeley CA 94720 USA; ^3^ School of Polymer Science and Engineering Center for Optoelectronic Materials and Devices The University of Southern Mississippi Hattiesburg MS 39406 USA; ^4^ College of Physics Guizhou University Guiyang 550025 P. R. China; ^5^ Institute of Polymer Optoelectronic Materials and Devices State Key Laboratory of Luminescent Materials and Devices South China University of Technology Guangzhou 510640 P. R. China; ^6^ College of Big Data and Information Engineering Guizhou University Guiyang 550025 P. R. China; ^7^ Materials Sciences Division Lawrence Berkeley National Laboratory One Cyclotron Road Berkeley CA 94720 USA

**Keywords:** charge carrier transport, green solvent, hybrid building block, semiconducting polymers, solution processability

## Abstract

The development of semiconducting polymers with good processability in green solvents and competitive electrical performance is essential for realizing sustainable large‐scale manufacturing and commercialization of organic electronics. A major obstacle is the processability‐performance dichotomy that is dictated by the lack of ideal building blocks with balanced polarity, solubility, electronic structures, and molecular conformation. Herein, through the integration of donor, quinoid and acceptor units, an unprecedented building block, namely TQBT, is introduced for constructing a serial of conjugated polymers. The TQBT, distinct in non‐symmetric structure and high dipole moment, imparts enhanced solubility in anisole—a green solvent—to the polymer TQBT‐T. Furthermore, PTQBT‐T possess a highly rigid and planar backbone owing to the nearly coplanar geometry and quinoidal nature of TQBT, resulting in strong aggregation in solution and localized aggregates in film. Remarkably, PTQBT‐T films spuncast from anisole exhibit a hole mobility of 2.30 cm^2^ V^‐1^ s^‐1^, which is record high for green solvent‐processable semiconducting polymers via spin‐coating, together with commendable operational and storage stability. The hybrid building block emerges as a pioneering electroactive unit, shedding light on future design strategies in high‐performance semiconducting polymers compatible with green processing and marking a significant stride towards ecofriendly organic electronics.

## Graphical abstract

A novel and groundbreaking three‐in‐one hybrid strategy through covalently integrating donor, quinoid, and acceptor is introduced to design semiconducting polymers with high polarity, nonsymmetric structure and backbone coplanarity, giving rise to good processibility in green solvent, exceptional charge carrier transport property, and noteworthy device stability.

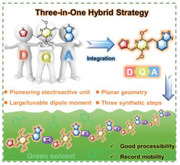

## 1 Introduction

Semiconducting polymers have attracted widespread research interest for their distinctive merits of mechanical flexibility, tunable optoelectronic property and cost‐effectiveness for large‐area manufacturing.^[^ ^1^, ^2^, ^3^, ^4^, ^5^, ^6^ ^]^ Recent decades have witnessed the significant progress of semiconducting polymers and their relevant applications in organic field‐effect transistors (OFETs),^[^ ^7^, ^8^ ^]^ organic photovoltaics (OPVs)^[^ ^9^, ^10^, ^11^, ^12^, ^13^ ^]^ and other organic electronic devices.^[^ ^14^, ^15^, ^16^ ^]^ Despite this progress, a major challenge persists: most high‐performing semiconducting polymers rely on toxic halogenated solvents such as chlorobenzene (CB), chloroform (CF) and *ortho*‐dichlorobenzene (o‐DCB) for processing, posing serious health and environmental concerns^[^ ^17^, ^18^, ^19^ ^]^ that limit sustainable large‐scale manufacturing and future commercialization of organic electronics.^[^ ^20^ ^]^ In consideration of low health hazard, high safety and small environmental impact, a high overall quantitative sustainability score (G) is desirable for ideal processing solvents according to an online tool proposed by Edman et al. based on GlaxoSmithKline (GSK) solvent sustainability guide.^[^ ^21^ ^]^ Moreover, on the basis of “Globally Harmonized System of Classification and Labelling of Chemicals (GHS)”^[^ ^22^ ^]^ of the United Nations and material safety data sheets (MSDSs), ideal solvent should not have the symbols of GHS05 (corrosive), GHS06 (toxic), GHS07 (harmful), GHS08 (health hazard), or GHS09 (environmental hazard).^[^ ^22^, ^23^ ^]^ Water, alcohols, and anisole are recommended in the solvent selection guides by CHEM21, Pfizer and Sanofi, which, thereby, are defined as green solvents.^[^ ^24^, ^25^, ^26^ ^]^ To address this challenge, developing polymers processable in green solvents becomes essential.^[^ ^27^, ^28^, ^29^ ^]^ However, this effort often results in a compromise between solubility and charge transport properties, creating a “Goldilocks zone” dilemma that impacts device performance when switching solvents.^[^ ^30^, ^31^, ^32^, ^33^ ^]^

**Figure 1:**
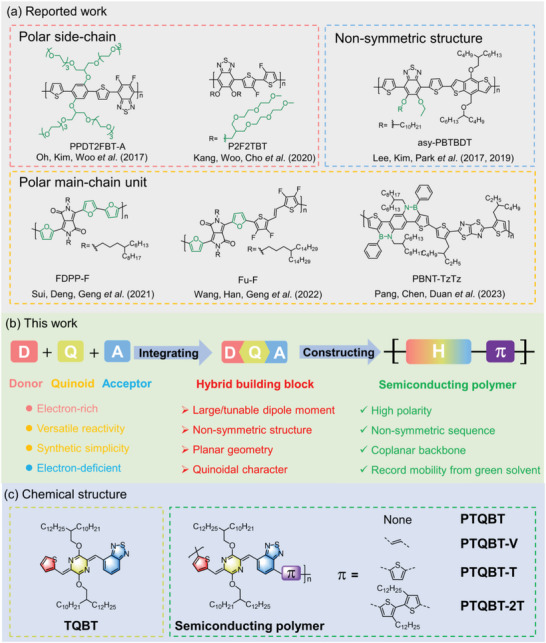
a) Reported works about the utilization of polar side‐chains, polar conjugated ring systems and non‐symmetric structures to increase the solubility of semiconducting polymers in green solvents, i.e., water, ethanol or anisole. b) Schematic illustration of the concept for designing hybrid building block to construct high‐performing green solvent‐processable semiconducting polymer in this work. c) Chemical structures of hybrid building block and corresponding semiconducting polymers.

In general, structural modifications for solubility concerns should avoid compromising the coplanarity of the conjugated ring system or interchain interactions in order to ensure efficient charge transport in conjugated polymers.^[^ ^34^, ^35^, ^36^, ^37^, ^38^ ^]^ Additionally, to enhance polymer solubility in green solvents, polymers should be designed with matching polarity with the solvent according to the principle of “like‐dissolves‐like.”^[^ ^23^, ^39^ ^]^ Strategies like installing polar side chains onto the polymer backbone have been explored to increase solubility in water or alcohols (**Figure** **1**a).^[^ ^40^ ^]^ However, the charge mobility of polymers bearing polar side chains was only around 0.1 cm^2^ V^‐1^ s^‐1^, which also encountered stability issues due to increased hygroscopicity.^[^ ^41^, ^42^ ^]^ Recent advancements include the incorporation of high dipole moment B─N bonds into the polymer backbone, as demonstrated by Duan and co‐workers, to increase the polarity of PBNT‐TzTz polymers and achieve good processability in food additive anisole,^[^ ^23^ ^]^ leading to a record‐high efficiency in green solvent‐processable OPV devices (Figure 1a).^[^ ^39^ ^]^ Furthermore, furan, a larger dipole moment unit than its analogue thiophene, was utilized by Geng and co‐workers to construct furan‐flanked diketopyrrolopyrrole (DPP) moiety. Polymers based on such moieties displayed sufficient solubilities in anisole (Figure 1a). Corresponding OFET devices fabricated from anisole showed charge mobilities up to 1.30 cm^2^ V^‐1^ s^‐1^, the highest value for spuncast semiconducting polymer processed from green solvents.^[^ ^43^ ^]^ Under optimized deposition conditions involving a bar‐coating method, those polymers delivered improved mobilities of up to 3.50 cm^2^ V^‐1^ s^‐1^.^[^ ^44^ ^]^ The above results indicate that increasing polarity of polymers via the installation of polar units is a viable approach to obtain high‐performing, green solvent‐processable semiconducting polymers.^[^ ^23^ ^]^ However, these examples primarily employ symmetric building blocks, which may partly cancel out the overall dipole moment and limit the overall increase in polymer polarity. To remedy this, nonsymmetric building blocks have been employed in conjugated polymers to lower the symmetry. Regio‐irregular polymers with a random sequence of non‐symmetric unit typically deliver disturbed intermolecular interactions, decreased interchain aggregations and lower thin film crystallinities in comparison to their regio‐regular counterparts, leading to an improved solubility.^[^ ^45^ ^]^ Park and co‐workers have demonstrated that introducing non‐symmetric moiety into polymer backbone (Figure 1a)^[^ ^23^, ^46^ ^]^ could render increased solubility in green solvents such as the food additive 2‐methylanisole, resulting in high efficiency perovskite solar cells, yet their use in high mobility transistors still remain to be demonstrated.^[^ ^47^ ^]^ Aside from these emerging efforts, creating innovative non‐symmetric building blocks with high dipole moments and planar conformation is desirable but remains largely underexplored in constructing high performance semiconducting polymers compatible with green processing.

The incorporation of quinoidal (Q) moiety into extended conjugated ring systems, alongside classic donor‐acceptor (D‐A) systems, has found widespread applications in the design and synthesis of conjugated polymers.^[^ ^48^, ^49^, ^50^, ^51^, ^52^, ^53^ ^]^ Properly chosen quinoidal units can lead to highly planar and rigid conjugated skeleton because of the effective minimization of bond length alternation, endowing the resulting polymer with impressive semiconducting properties.^[^ ^54^, ^55^ ^]^ Quinoidal building blocks with symmetric flanking units such as phenyl, thiophene and thiophene‐fused heteroaromatic moiety are more well known,^[^ ^56^, ^57^ ^]^ while non‐symmetrically substituted quinoids with contrasting electronic side groups remains unexplored due to synthetic challenges and stability concerns.^[^ ^58^ ^]^ The development of the stable and versatile *para*‐azaquinodimethane (*p*‐AQM) motif, pioneered by us in 2017, offers a promising path forward towards non‐symmetrically functionalized polar electronic components for green processible conjugated polymers.^[^ ^59^, ^60^, ^61^, ^62^, ^63^, ^64^, ^65^, ^66^ ^]^ Herein, a hybrid building block, namely TQBT, is designed to integrate donor, quinoid and acceptor moieties into one non‐symmetric structure, featuring a large and tunable dipole moment, a planar geometry and intrinsic quinoidal characters (Figure 1b). By copolymerizing with different comonomers, a series of conjugated polymers with various sizes of repeating units and tunable polarities can be produced. Thanks to the synergistic effects of high polarity, nonsymmetric sequence and the backbone coplanarity, a TQBT‐based polymer with thiophene as the comonomer unit show good solubility in anisole^[^ ^33^, ^39^ ^]^ while forming localized aggregates in the solid state to ensure efficient charge transport. The green solvent processibility is commensurate with excellent charge transport properties, delivering an impressive hole mobility of 2.30 cm^2^ V^‐1^ s^‐1^ and commendable device stability.

## 2 Results and Discussion

### 2.1 Molecular Design

Nonsymmetric donor–acceptor (D–A) structure usually translates to a large dipole moment because of the strong intramolecular electron push–pull effect,^[^ ^67^ ^]^ which might be utilized for producing high‐polarity semiconducting polymers.^[^ ^68^, ^69^ ^]^ However, the synthesis of nonsymmetric D–A monomers is non‐trivial due to the constraints of different reactivities between donor and acceptor units.^[^ ^70^ ^]^ In addition, directly linking donor and acceptor units might render large dihedral angles and twisted conformations due to steric hindrance, which will adversely impact π–π interchain packing in the corresponding polymers.^[^ ^71^, ^72^ ^]^ Furthermore, it is generally known that increasing the distance between the centers of positive and negative charges will increase the dipole moment.^[^ ^73^ ^]^ In our design, thiophene (T) and benzothiadiazole (BT) are selected as the donor and acceptor moieties, respectively, which are simple and attractive building blocks for high‐performance semiconducting polymers. *p*‐AQM is inserted between thiophene and benzothiadiazole moieties, giving rise to a hybrid building block incorporating all three donor, quinoid and acceptor moieties (Figure 1c). A highly coplanar conformation is expected due to the presence of S…N interaction between thiophene and *p*‐AQM^[^ ^62^ ^]^ and the torsion‐free CH…N arrangement between benzothiadiazole and *p*‐AQM.

A series of conjugated polymers with varying length of repeat units and tunable polarities was designed to investigate the basic optoelectronic properties of TQBT polymers and evaluate their green solvent‐processability, including a homopolymer, namely PTQBT, and three alternating copolymers, namely PTQBT‐V, PTQBT‐T and PTQBT‐2T (Figure 1c). The commonly used vinyl group and oligothiophene units were selected because of their great potential in constructing high mobility conjugated polymers and different molecular size/length, which would additionally endow the resulting polymers with tunable molecular polarities.^[^ ^74^, ^75^, ^76^ ^]^ To ensure sufficient solubility in solvents, additional octyl sidechains were introduced onto the bithiophene unit of PTQBT‐2T. The TQBT‐based polymers are expected to display large and tunable molecular polarity and coplanar chain conformation, ultimately giving rise to good green solvent processibility and excellent charge carrier mobility.

### 2.2 Theoretical Calculations

**Figure 2:**
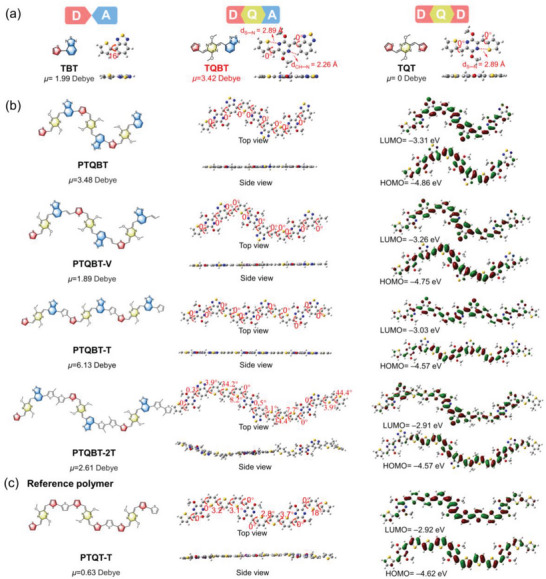
Optimized geometries and dipole moments of a) representative D–A, D–Q–A, and D–Q–D type building blocks and b) trimeric segments of four corresponding D–Q–A‐based polymers and c) the reference analogous polymer PTQT‐T.

To support the above molecular design rationale, density functional theory (DFT) calculations were carried out employing Gaussian 09 at the B3LYP/6‐311G (d,p) level, with all the alkyl chains replaced with methyl groups for computational simplicity. As illustrated in **Figure** **2**a, the direct coupling of T with BT in the representative D–A building block TBT resulted in a dipole moment of 1.99 Debye and a twisted conformation with a dihedral angle of 16°, owing to the non‐symmetric nature and spatial repulsion effect between two adjacent subunits, respectively. Inserting the quinoidal *p*‐AQM in between the D–A moieties in TBT could endow the resulting non‐symmetric D–Q–A building block TQBT with a significantly increased dipole moment of up to 3.42 Debye. Moreover, the contact distances of S…N and CH…N are 2.89 Å and 2.26 Å, respectively, significantly shorter than their respective sum of the van der Waals radii of 3.35 and 2.86 Å.^[^ ^77^, ^78^ ^]^ These values indicate the existence of intramolecular S…N interaction and hydrogen bonding within TQBT unit, locking in a coplanar conformation with the dihedral angles of ≈0° between the thiophene/benzothiadiazole and *p*‐AQM moieties. The large dipole moment and planar geometry associated with TQBT are also present in other D–Q–A systems with different D/A combinations (Figure S1, Supporting Information). Such facile and effective regulation of the dipole moment by simply varying donor and/or acceptor structures is conductive to tune the polarity of the corresponding polymers and consequently solubility in green solvents. For example, replacing thiophene with furan or substituting benzothiadiazole with benzooxadiazole in TQBT resulted in new D–Q–A building blocks FQBT and TQBO, the dipole moments of which were altered to 4.01 and 6.02 Debye, respectively, significantly larger than that of their respective D–A building blocks (2.39 and 4.38 Debye, respectively, Figure S1, Supporting Information). In contrast, the dipole moment of TQT, the symmetric D–Q–D counterpart with a thiophene unit flanking on both sides of AQM, was 0 Debye, in accordance with its centrosymmetric nature. In addition, lowest unoccupied molecular orbital (LUMO) and highest occupied molecular orbital (HOMO) energy levels and the overall bandgap of TQBT were lowered in comparison to TQT, owing to the substitution of electron‐rich T with electron‐deficient BT (Figure S2, Supporting Information). Moreover, electrostatic potential (ESP) distributions on the molecular surfaces were also calculated. As shown in Figure S2 (Supporting Information), TQBT exhibited larger average ESP value relative to TQT, indicative of the stronger electron‐deficient nature of TQBT owing to the existence of BT moiety.

Considering the non‐symmetric nature of TQBT, the dimeric units of each of the four TQBT‐based polymers may have three possible regioisomers (Figure S3, Supporting Information). Except the head‐to‐head and tail‐to‐tail dimeric segments of the homopolymer PTQBT which have net zero dipole moment, the dipole moments of the three dimeric units for all the copolymers are not cancelled out, which can be attributed to their nonlinear conformation despite their almost coplanar backbones. As the different connectivity from the three isomers shows little impact on the coplanarity, only the head‐to‐tail trimeric segments of each of the polymers were modelled to evaluate the molecular polarity, backbone geometry and frontier molecular orbital distributions. As shown in Figure 2b, the dipole moments of the four trimers are in the range of 1.89–6.13 Debye, significantly larger than that of the reported polymers PBNT‐TzTz and FDPP‐F (Figure 1a and Figure S5, Supporting Information). The dipole moment of polymers based on FQBT and TQBO hybrid block with varied D/A combinations could be further increased to 8.33 and 13.43 Debye, respectively (Figure S6, Supporting Information). This result confirms that the hybrid building block is more effective in increasing the dipole moments of polymers than previous methods. To further corroborate this, trimers with different TQBT orientations in the copolymer PTQBT‐T were also modeled. As illustrated in Figure S4 (Supporting Information), the dipole moments of those four trimers were in the range of 6.09−6.39 Debye, which were comparable to each other and irrelevant to the sequence. The results revealed that despite the random sequence and orientation of TQBT in the polymer backbone, the dipole moment of the nonsymmetric TQBT units does not cancel out but instead will translate into greater polarity in the resulting polymers when coupled with proper comonomer units. With thiophene as the comonomer unit, the trimer of PTQBT‐T possessed the highest dipole moment among the TQBT‐based series, which far exceeded the TQT‐based analogous polymer PTQT‐T (*μ* = 0.63 Debye) (Figure 2c). In addition, with the exception of PTQBT‐2T which contains steric alkyl groups on the bithiophene unit, all the TQBT‐based polymers exhibit nearly coplanar backbone conformation with dihedral angles between all neighboring subunits of 0°, whereas increased dihedral angle and twisted geometry are observed in their TQT‐based counterparts (Figure S7, Supporting Information). Moreover, almost all of the calculated LUMO and HOMO energy levels of TQBT‐based polymers were lower than their respective TQT‐based analogous polymers (Figure 2c and Figure S7, Supporting Information), in full consistency with the experimental results (vide infra). The comparative analysis manifests that the hybrid TQBT building block cannot only endow the resulting polymers with increased polarity, but also lead to more planar backbone geometry and decreased energy levels.

**Figure 3:**
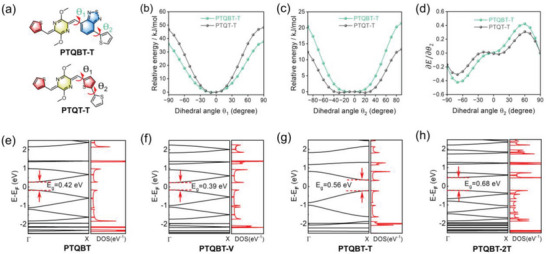
a) Theoretical mode for the calculation of torsional potential energy. Calculated torsional energy barrier as a function of b) dihedral angel (*θ*_1_) and c) dihedral angel (*θ*_2_). d) First derivative of calculated torsional energy barrier as a function of dihedral angel (*θ*_2_). Band structure and partial density of state (DOS) of e) PTQBT, f) PTQBT‐V, g) PTQBT‐T and h) PTQBT‐2T.

To probe the stable geometrical conformation and assess the backbone rigidity of TQBT‐based polymers, torsional potential energy dependence on the dihedral angles between *p*‐AQM and flanking BT/T (*θ*_1_), and between flanking BT/T and co‐monomer T (*θ*_2_) for PTQBT‐T and was calculated and compared against that of the analogue PTQT‐T (**Figures** **3**a–c and S8, Supporting Information). Despite a slightly increased energy barrier for PTQT‐T upon the rotation of θ_1_ (Figure 3b), PTQBT‐T displayed a substantially larger energy barrier than that of PTQT‐T upon the rotation of θ_2_ (Figure 3c). Moreover, as shown in Figure 3d, the two units defining the dihedral angel θ_2_ of PTQT‐T could rotate barrierlessly within a wide angle range from ‐30° to 30°, while that of PTQBT‐T exhibited a narrower range (‐10° to 10°). The results suggest that TQBT building block can impart resulting polymers with strengthened backbone rigidity, which would reduce the conformation disorder and increase the interchain interaction of polymers.^[^ ^79^, ^80^ ^]^ To validate this, the intermolecular binding energy for PTQBT‐T and PTQT‐T were further calculated based on their respective optimized dimers with the DFT‐D3 method.^[^ ^81^, ^82^ ^]^ As shown in Figure S9 (Supporting Information), the binding energy of PTQBT‐T was much smaller than that of PTQT‐T, which demonstrate the enhanced electrostatic interaction in PTQBT‐T and accords well with its enhanced intermolecular interactions and excellent charge carrier transport property (vide infra). Furthermore, effective hole masses (*m*_h_*) were calculated using Vienna ab initio simulation package (VASP) with Perdew‐Burke‐Ernzerhof (PBE) functional to evaluate intramolecular charge transport capability of TQBT‐based polymers.^[^ ^83^ ^]^ Band structures, partial densities of states (DOS) and calculated *m*_h_* were shown in Figure 3e–h, **Table** **1** and Figure S10, Supporting Information. PTQBT‐T possessed the smallest *m_h_* * of only 0.096 *m_e_* among all the polymers, while the *m*_h_*s of other polymers were relatively large and ranged from 0.101 to 0.141 *m*_e_. In the case of the reference polymer PTQT‐T, its *m*_h_* was larger than that of PTQBT‐T.^[^ ^66^ ^]^ The small *m*_h_* manifests efficient hole transport along the backbone of PTQBT‐T, in good accordance with its high charge carrier mobility measured from the corresponding OFET devices (vide infra).

**Table 1:** Summary of molecular weight, solubility, optical bandgap, electrochemical properties and effective hole masses of four TQBT‐based polymers and reference PTQT‐T.

Polymer	*M*_n_ ^a^ ^)^ [KDa]/*Ð*	Solubility [mg mL^−1^]		Solution		Film		*E*_g_ ^d)^ [eV]	HOMO^e)^ [eV]	LUMO^f)^ [eV]	*m*_h_^*^ [*m*_e_]^g)^
				*λ*_max1_ [nm]	*λ*_max2_ [nm]	*λ*_max1_ [nm]	*λ*_max2_ [nm]				
PTQBT	14.5/1.9	14.3^b)^	<0.01^c)^	666	–^h)^	687	–^h)^	1.29	‐5.02	‐3.73	0.101
PTQBT‐V	13.7/2.6	17.2^b)^	<0.01^c)^	709	–^h)^	743	–^h)^	1.33	‐5.16	‐3.83	0.107
PTQBT‐T	17.8/2.6	20.5^b)^	7.60^c)^	722	787	728	805	1.36	‐5.17	‐3.81	0.096
PTQBT‐2T	15.5/1.9	10.6^b)^	<0.01^c)^	607	–^h)^	642	–^h)^	1.50	‐5.27	‐3.77	0.141
PTQT‐T^i)^	38.9/2.0	23.1^b)^	<0.01^c)^	687	742	688	758	1.49	‐5.02	‐3.53	0.102

^a)^ Molecular weight measured by high temperature SEC;

^b)^ In chlorobenzene at 70 °C;

^c)^ In anisole at 70 °C;

^d)^ Optical bandgaps estimated from the absorption onset;

^e)^ Measured by cyclic voltammetry;

^f)^ Calculated based on film optical bandgap and electrochemical HOMO levels;

^g)^ Effective hole mass (*m*_h_*) extracted from theoretical calculations. *m_e_* represents the mass of an electron;

^h)^ Not available;

^i)^ Reported previously.^[^ ^62^ ^]^

The theoretically computed bandgaps follow the trend of PTQBT‐2T > PTQBT‐T > PTQBT > PTQBT‐V, in close agreement with experimental results (vide infra) where increased number of thiophene moieties in each repeat unit tends to confer enlarged bandgap. This unique trend of bandgap is opposite to that in conventional D–A polymers but consistent with that in quinoidal‐aromatic polymers,^[^ ^62^, ^64^ ^]^ which confirms the quinoidal character of the hybrid TQBT building block. As shown in Figure S10 (Supporting Information), the extent of bond length alternation (BLA) of the segments between adjacent *p*‐AQM unit in all polymers except PTQBT‐V increases with increasing number of thiophene units, which is in good accordance with our previous findings and can rationally support the bandgap trend.^[^ ^84^ ^]^

### 2.3 Synthesis of TQBTs and TQBT‐Based Polymers

**Scheme 1:**
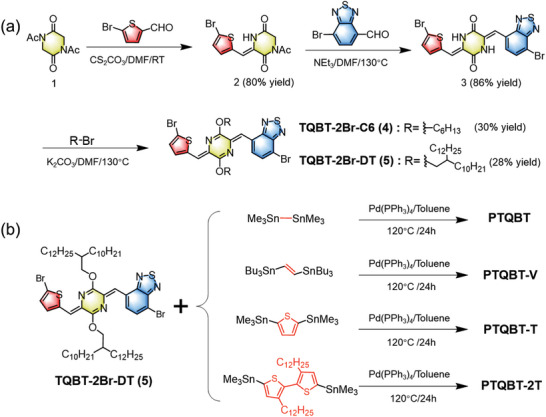
Synthesis of a) the dibromo‐TQBT‐based monomers and b) the TQBT‐based polymers.

The synthetic routes of TQBT‐based monomers and corresponding polymers are illustrated in **Scheme** **1**. Detailed synthesis and characterization are described in Supporting Information. The key dibromo monomers were readily prepared in only three steps staring from the commercially available **1** with a decent overall yield. Monobromide **2** was obtained via the 1:1 Knoevenagel condensation between 1,4‐diacetyl‐2,5‐diketopiperazine (**1**) and 5‐bromothiophene‐2‐carbaldehyde in 80% yield, which was further reacted with 7‐bromobenzothiadiazole‐4‐carbaldehyde through another 1:1 Knoevenagel condensation to yield dibromo intermediate **3** in a high yield of 86%. The subsequent alkylation of **3** with 1‐bromohexane or 11‐(bromomethyl) tricosane gave rise to TQBT‐based monomer TQBT‐2Br‐C6 (**4**) in 30% yield or TQBT‐2Br‐DT (**5**) in 28% yield, respectively. Four polymers were synthesized by Stille copolymerization between **5** and the corresponding distannylated monomers at 120 °C for 24 h and purified subsequently by successive Soxhlet extraction. Reprecipitating the chloroform extracted fractions resulted in the target products. The molecular weights of four polymers were measured by high temperature size exclusion chromatography (SEC) in 1,2,4‐trichlorobenzene at 140 °C with polystyrene as the standard. As shown in Table 1, the four polymers showed comparable number‐average molecular weight (*M*_n_) and polydispersity index (PDI). Thermogravimetric analysis (TGA) was conducted to evaluate the thermal properties of polymers. The decomposition temperature (*T*_d_) at a 5% weight loss was located in the range of 376.3–390.0 °C, indicating good thermal stabilities for all the polymers (Figure S11, Supporting Information). It is worth noting that those TQBT‐based polymers are regio‐irregular with random sequences of TQBT units in the backbone. Inspired by the prospects of how regioregularity of conjugated polymers impacts the intrinsic properties, film morphologies and performances of the resulting devices,^[^ ^45^ ^]^ strategies that enable the synthesis of regio‐regular TQBT‐based polymers are currently being pursued in our lab.

### 2.4 Structural Characterization

**Figure 4:**
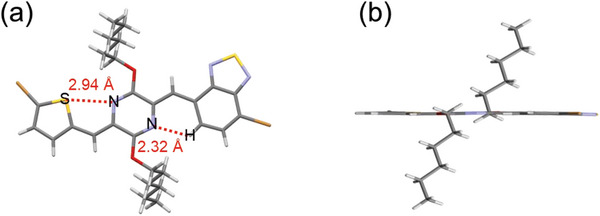
X‐ray structure of **4**. Capped‐stick representation of a) top view and b) side view.

Proton and carbon nuclear magnetic resonance (NMR) and single‐crystal X‐ray analysis were conducted to confirm the chemical structure of TQBT. As shown in Supporting Information, the ^1^H and ^13^C NMR spectra of **4** and **5** are in full consistency with the structure depicted in Scheme 1. In addition, the single crystal structure of **4** was illustrated in **Figure** **4**, which provided clear evidence of the non‐symmetric structure and coplanar geometry. The contact distances of S…N and CH…N are 2.94 Å and 2.32 Å, respectively, similar to the theoretically calculated result. The results undoubtedly reveal the presence of intramolecular S…N interaction and CH…N hydrogen bonding in TQBT, which led to the almost coplanar geometry (Figure 4b). The dihedral angle between flanking thiophene/benzothiadiazole and central *p*‐AQM groups is 2.1° and 0.5°, respectively, notably smaller than those (≈3°) in the TQT unit (Figure S12, Supporting Information),^[^ ^62^ ^]^ indicating that the combination of donor, quinoid and acceptor unit gives rise to a more flattened geometry.

### 2.5 Solubility Test

**Figure 5:**
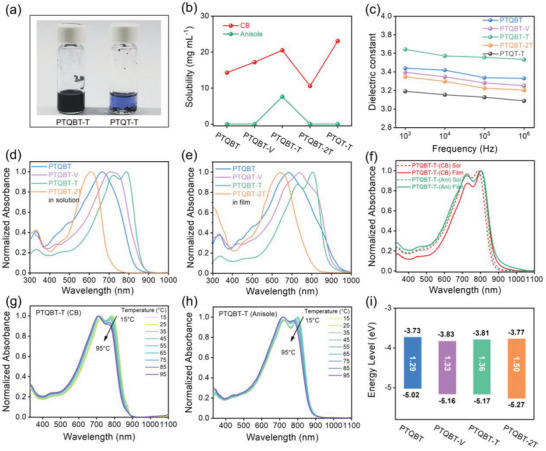
a) Photographs of PTQBT‐T and PTQT‐T in anisole at 70 °C. b) The solubilities of polymers in chlorobenzene and anisole in 70 °C. c) The relative dielectric constants of polymers at different frequencies. Normalized UV–vis absorption spectra of TQBT‐based polymers d) in chlorobenzene and e) thin film at room temperature. f) Comparison of UV–vis absorption spectra of PTQBT‐T in chlorobenzene and anisole, and its thin film from chlorobenzene and anisole at room temperature. The evolution of UV–vis absorption spectra of PTQBT‐T in g) chlorobenzene and h) anisole during heating. i) Diagram of energy levels of TQBT‐based polymers.

The solubilities of those four polymers in the commonly used chlorinated solvent CB and the green solvent anisole were evaluated. For comparison, solubility test of the TQT‐based reference polymer PTQT‐T was also conducted. As shown in Figure S13 (Supporting Information), all TQBT‐based polymers and PTQT‐T were well soluble in CB at 70 °C. However, when moving from CB to anisole, only PTQBT‐T could be readily dissolved in anisole at 70 °C, while the rest of polymers including PTQT‐T were insoluble in anisole even after heat treatment (**Figures** **5**a and S14, Supporting Information). The quantitative solubility of each polymer in CB and anisole was further determined^[^ ^85^ ^]^ and shown in Figure 5b, Figure S15 (Supporting Information), and Table 1. The solubilities of four TQBT‐based polymers in 70 °C CB ranged from 10.6 to 20.5 mg mL^−1^, which followed the order of PTQBT‐T > PTQBT‐V > PTQBT >PTQBT‐2T. The reference polymer PTQT‐T showed a slightly higher solubility of 23.1 mg mL^−1^ in comparison to PTQBT‐T. In sharp contrast, in anisole at 70 °C, PTQBT‐T showed an appreciable solubility of 7.60 mg mL^−1^ while the solubilities of reference PTQT‐T and the rest of TQBT‐based polymers were lower than < 0.01 mg mL^−1^. It has been suggested that higher polarity of polymer corresponds to higher dielectric constant, which would contribute to an increased solubility in polar solvents.^[^ ^39^ ^]^ The relative dielectric constant (*ε*_r_) of four TQBT‐based polymers and the reference PTQT‐T were probed in the frequency range from 1 × 10^3^ to 1 × 10^6^ Hz by the capacitance−voltage (*C−V*) measurement. As depicted in Figure 5c and Table S1 (Supporting Information), PTQBT‐T has the largest *ε*_r_ value of 3.62−3.57, in good accordance with its highest solubility in polar solvent anisole. PTQBT, PTQBT‐V and PTQBT‐2T shared similar *ε*_r_ values (ca. 3.40−3.38), which were smaller than that of PTQBT‐T but substantially larger than that of the reference polymer PTQT‐T (3.19−3.15). The order of dielectric constants of all polymers is in good agreement with the trend of their dipole moments, confirming the higher polarity of TQBT‐based polymers and the excellent capacity of TQBT unit in constructing green solvent‐processable conjugated polymers based on a rational molecular design.

### 2.6 Optical and Optoelectronic Properties

The UV–vis absorption spectra of the four TQBT‐based polymers in dilute CB and thin films at room temperature (RT) are shown in Figure 5d,e, respectively, and summarized in Table 1. The absorption spectrum of monomer **5** TQBT‐2Br‐DT was also measured for comparison. As shown in Figure S16 (Supporting Information), TQBT‐2Br‐DT displayed a substantially red‐shifted absorption in comparison with its analogous TQT‐based monomer TQT‐2Br‐DT, as expected from its stronger electron‐deficient characters. Moreover, TQBT‐2Br‐DT exhibited higher oxidation potential than TQT‐2Br‐DT judging from their CV curves (Figure S16, Supporting Information), indicating its lower HOMO level as predicted by theoretical calculations. For polymers, PTQBT‐2T showed obviously blue‐shifted and featureless absorption in CB when compared to other three polymers, owing to the steric‐induced non‐coplanarity and decreased interchain interactions and the lessened quinoidal character. In sharp contrast, pronounced 0−0 and 0−1 peaks with *A*_0−0_/*A*_0−1_ ratio of above 1 were observed in PTQBT‐T, indicating strong interchain aggregation and ordered interchain packing in solution.^[^ ^86^, ^87^, ^88^, ^89^ ^]^ As for PTQBT and PTQBT‐V, they exhibited a main absorption peak together with a weak shoulder peak at longer wavelength, the position of which was located between that of PTQBT‐2T and PTQBT. When shifting from solution to thin film, the absorption spectra of all polymers but PTQBT‐T redshifted significantly, implying tightened interchain stacking in the solid state. In the case of PTQBT‐T, despite an increased *A*_0−0_/*A*_0−1_ ratio and a slight bathochromic shift, PTQBT‐T showed similar absorption spectra to that in solution, which suggested that strong pre‐aggregation was established in the solution state and became more ordered during solidification. The strong and ordered interchain stacking in PTQBT‐T is presumably associated with its highly planar geometry, rigid backbone and large molecular dipole moment, which are expected to facilitate charge carrier transport.^[^ ^90^, ^91^ ^]^ In comparison, absorption spectra of PTQBT‐T in dilute anisole solution and thin film spuncast from anisole were also acquired. PTQBT‐T showed very similar but slightly red‐shifted absorption spectra with an identical *A*_0−0_/*A*_0−1_ ratio in anisole relative to that in CB solution (Figure 5f). The absorption spectra of PTQBT‐T thin film spuncast from anisole was also almost identical to that in anisole solution, both of which were comparable to the CB processed thin films except a slightly decreased *A*_0−0_/*A*_0−1_ ratio. Variable‐temperature absorption spectra in both dilute CB and anisole solutions were acquired to gain further insight into the interchain aggregation behavior of PTQBT‐T in different solvent systems. As shown in Figure 5g,h, the absorption spectra in both CB and anisole solutions exhibited gradual blue‐shift together with a slight decrease in the intensity of 0−0 peak upon heating the solutions from 15 °C to 95 °C, confirming the strong interchain aggregation of PTQBT‐T in both solutions. However, despite identical *A*_0‐0_/*A*_0‐1_ ratios were observed at 15 °C, the *A*_0‐0_/*A*_0‐1_ ratio in CB and anisole solution was decreased to 0.91 and 0.96, respectively. What is more, filtration experiments were also carried out to probe the size of aggregate in CB and anisole.^[^ ^92^, ^93^ ^]^ As shown in Figure S17 (Supporting Information), PTQBT‐T in CB showed larger amounts of small aggregate than in anisole, while the amounts of large aggregate became more in anisole, evidencing that PTQBT‐T possessed stronger interchain aggregation in anisole than in CB. The comparative results suggest that anisole not only increased the interchain entanglement in the solution state, but also slightly disrupted the ordering of interchain packing in polymer thin films (vide infra).

The optical bandgaps of polymers estimated from thin film absorption were in the range of 1.29−1.50 eV, which followed the order of PTQBT‐2T > PTQBT‐T > PTQBT‐V > PTQBT. The results confirmed the quinoidal character of this hybrid building block TQBT and was in full consistency with computationally predicted trend of bandgap and BLA described above. CV measurements were conducted to probe the HOMO and LUMO energy levels of the TQBT‐based polymers (Figure S18, Supporting Information). As presented in Figure 5i and Table 1, the HOMO energy levels were in the range of −5.02 to −5.27 eV, while the LUMO energy levels were comparable for those polymers. The results indicated that the HOMO energy level lowered with increasing length of repeat unit in the conjugated polymer, also consistent with the trend observed in *p*‐AQM‐based quinoidal−aromatic conjugated polymers.^[^ ^61^, ^62^ ^]^ Furthermore, both HOMO and LUMO energy levels of TQBT‐based polymers obtained from either calculation or experiment were down‐shifted compared to the TQT‐based analogue, due to the stronger electron‐withdrawing property of TQBT unit (Figure S19, Supporting Information).

### 2.7 OFET Fabrication and Characterization

**Figure 6:**
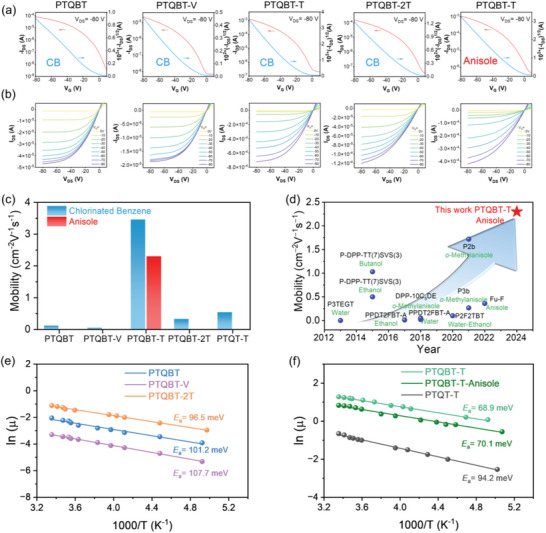
Typical a) transfer and b) output characteristics of annealed OFETs based on four TQBT‐based polymers spuncast from chlorobenzene or anisole. c) Comparison of maximum hole mobilities of the four TQBT‐based polymers. d) Comparison chart of reported polymer OFETs spuncast from green solvents. Arrhenius plots of mobility against temperature of e) PTQBT, PTQBT‐V and PTQBT‐2T and f) PTQBT‐T, PTQT‐T and PTQBT‐T processed from anisole.

To evaluate charge carrier transport property of TQBT‐based polymers, bottom‐gate top‐contact (BGTC) OFET devices were fabricated. Polymer films were spuncast from CB or anisole when applicable and then annealed at the optimized temperatures of 150 or 200 °C. Representative transfer and output characteristics were depicted in **Figures** **6**a,b and S20 (Supporting Information), and device performance was summarized in **Table** **2**, Table S2 and Figure S21 (Supporting Information). For comparison, the relevant parameters of analogue PTQT‐T were also included. All annealed OFET devices displayed typical p‐type transport behavior, correlating well with the relatively shallow HOMO energy levels of those TQBT‐based polymers. Large off‐currents were observed in OFET devices, presumably ascribed to the oxidative O_2_ doping.^[^ ^94^ ^]^ With CB as the processing solvent, PTQBT‐T delivered the highest hole mobility of 3.46 cm^2^ V^‐1^ s^‐1^ among four polymers, while the hole mobilities of other three TQBT‐based polymers were in the range of 0.05−0.33 cm^2^ V^‐1^ s^‐1^. Notably, the hole mobility of PTQBT‐T was about one order higher than its analogous polymer PTQT‐T (0.54 cm^2^ V^‐1^ s^‐1^),^[^ ^62^ ^]^ demonstrating the superiority of hybrid TQBT building block in achieving high charge transport properties (Figure 6c). As aforementioned, PTQBT‐T possessed good solubility in green solvent anisole solution owing to its relatively large polarity, allowing the fabrication of relevant electronic devices via green processing. Therefore, PTQBT‐T‐based OFET devices were further fabricated by spin‐coating with anisole as the processing solvent. Impressively, a remarkable maximum hole mobility of 2.30 cm^2^ V^‐1^ s^‐1^ with a high reliability factor of 80% was achieved (Figure 6d, Figure S23 and Table S3, Supporting Information). Notably, the mobilities of all TQBT‐based polymers display a relatively low gate voltage dependence (i.e., small mobility variations) in the range of −20 to −80 V, which are consistent with their reliability factors observed in OFET devices and indicate high confidence level of the device measurement (Figure S22, Supporting Information). To the best of our knowledge, the hole mobility represents the highest value for semiconducting polymers processed from green solvents using the conventional spin‐coating method, and is comparable to two exceptional examples exhibiting higher hole mobilities of 2.7 and 3.5 cm^2^ V^‐1^ s^‐1^, which were obtained by the non‐conventional pray‐coating and bar‐coating methods, respectively.^[^ ^44^, ^95^ ^]^

**Table 2:** OFET device performance of annealed TQBT‐based polymers and the reference TQT‐based analogous polymer.

Polymer	Solvent	*µ*_h, max_ [*µ*_h, avg_]^a^ ^)^ [cm^2^ V^‐1^ s^‐1^]	*V*_th_ [V]	*I*_on/off_	*S* [V per dec]	γ^b)^ [%]
PTQBT	CB	0.12 (0.084±0.021)	‐3	10^4^–10^5^	‐5	89
PTQBT‐V	CB	0.05 (0.042 ± 0.006)	‐9	10^3^–10^4^	‐10	80
PTQBT‐T	CB	3.46 (2.93 ± 0.29)	‐11	10^3^–10^4^	‐9	82
PTQBT‐2T	CB	0.33 (0.28 ± 0.03)	‐10	10^3^–10^4^	‐11	83
PTQT‐T^c)^	o‐DCB	0.54 (0.47 ± 0.074)	‐14	5×10^5^	–^d)^	–^d)^
PTQBT‐T	Anisole	2.30 (2.01 ± 0.22)	‐5	10^3^–10^4^	‐13	80

^a)^ Maximum mobility under optimized annealing conditions. Average mobilities were calculated based on 10 independent devices and listed in parentheses;

^b)^ Reliability factor;

^c)^ Reported previously;^[^ ^62^ ^]^

^d)^ Not available.

Temperature‐dependent mobility measurements were carried out to better understand the difference in the mobility of those OFET devices and the origin of the efficient charge carrier transport in PTQBT‐T. As depicted in Figure 6e,f, all OFET devices exhibited a positive relationship between temperature and mobility, which were commonly observed in high mobility conjugated polymers and indicated a thermal‐activated hopping transport mechanism.^[^ ^96^, ^97^ ^]^ The temperature dependence of hole mobility was fitted with Arrhenius equation, from which the activation energy (*E*_a_) could be extracted to be in the range of 96.5−107.7 meV for PTQBT, PTQBT‐V and PTQBT‐2T (Figure 6e). In contrast, the *E*_a_ of PTQBT‐T based OFET devices were only 68.9 and 70.1 meV for those processed from CB and anisole, respectively, lower than that of all other TQBT polymers and the reference polymer PTQT‐T (94.2 meV), which correlated well with its most efficient charge transport property (Figure 6f).^[^ ^98^, ^99^ ^]^

**Figure 7:**
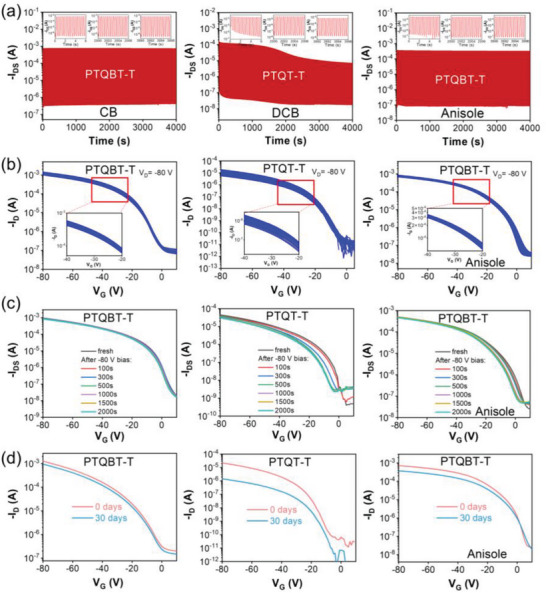
a) On–off cyclic tests (9000 cycles) by applying *V*_G_ of −80 and 0 V at *V*_D_ of −80 V, b) repeatability of transfer curves (30 cycles) by applying *V*_D_ of −80 V, c) bias stress tests by applying continuous bias voltage of −80 V for up to 2000 s and d) transfer curves before and after storing in ambient air for 30 d of OFET devices based on PTQBT‐T and PTQT‐T.

Inspired by the excellent charge transport properties of TQBT‐based polymers, the OFET devices were tested to investigate the operational stability under bias and the storage stability in ambient air, both of which are crucial for practical usage.^[^ ^100^, ^101^ ^]^ As indicated in **Figures** **7**a and S24a (Supporting Information), the OFET devices based on CB‐processed TQBT polymers displayed negligible fluctuations in both on‐current and off‐current during on‐off cyclic tests up to 9000 cycles and 4000 s, reflecting impressive operational stability. In contrast, the operational stability of OFETs based on *o*‐DCB‐processed PTQT‐T thin film was considerably worse, as evidenced by obvious drop in both on‐current and off‐current and decreased on/off ratio. The comparative results indicated that the hybrid TQBT building block could endow the resulting polymers with better operational stability than its analogous TQT unit. Similar trends were observed regarding the repeatability of transfer characteristics and the bias stress stability, where TQBT‐based polymers outperformed the TQT‐based analogous polymer (Figure 7b,c and Figure S24b,c, Supporting Information). Moreover, TQBT‐based polymers also showed better storage stability than PTQT‐T, as judged from the smaller shift of the device transfer curves than PTQT‐T (Figure 7d and Figure S24d, Supporting Information) after being storing in ambient air for one month. The superior storage stability of TQBT‐based polymers is presumably due to their lower HOMO and LUMO energy levels. The operational and storage stability of anisole‐processed device was also good, despite minimal change of transfer characteristics during bias stress test and storage stability evaluation (Figure 7 and Figure S24, Supporting Information).

### 2.8 Thin Film Microstructures and Morphologies

**Figure 8:**
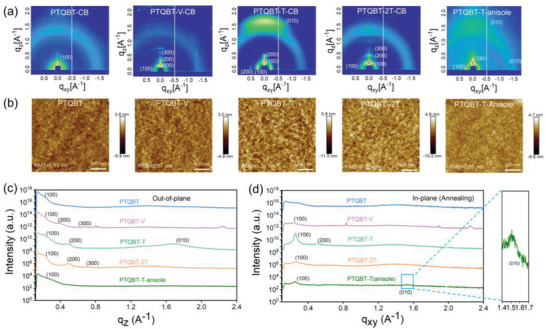
a) 2D GIWAXS images and b) AFM images of annealed polymer films. 1D GIWAXS plots along c) out‐of‐plane and d) in‐plane directions for annealed polymer films.

Thin film microstructures of those polymer films were investigated by grazing‐incidence wide‐angle X‐ray scattering (GIWAXS) measurement to correlate with their charge transport properties (**Figures** **8** and S25, Supporting Information).^[^ ^102^ ^]^ All annealed thin films spuncast from CB exhibited distinct diffraction peaks in out‐of‐plane (OOP) and in‐plane (IP) directions except for PTQBT, indicative of crystalline nature of those three polymer thin films (Figure 8a). More specifically, PTQBT showed a weak OOP (100) diffraction peak, which implied substantially disordered microstructure of its thin film and agreed well with its inferior hole mobility. As for PTQBT‐V and PTQBT‐2T, characteristic OOP (h00) diffraction peaks (h up to 3) were observed, suggesting that the films were more ordered and predominantly adopted edge‐on orientation. The crystallinity of PTQBT‐2T was higher than that of PTQBT‐V, as evidenced by the sharper diffraction peaks. In clear contrast, PTQBT‐T adopted bimodal crystallite orientations, evidenced by the presence of strong OOP (010) diffraction peak, IP and OOP (h00) diffraction peaks (h up to 2) (Figure 8c,d). The strong peak intensity suggested the highly‐ordered crystalline feature of PTQBT‐T, which was in good accordance with its excellent hole transport property.

In the case of annealed PTQBT‐T film processed from anisole, the film displayed weak diffraction peaks together with the (100) Debye ring, revealing a somewhat disordered packing structure without long‐range ordering.^[^ ^103^ ^]^ Moreover, (100) and (010) diffraction peaks were present in both OOP and IP directions, indicative of the coexistence of face‐on and edge‐on orientation. The emergence of IP (010) diffraction peak suggested the presence of IP π−π stacks between conjugated backbones, which might be promoted by strengthened interchain aggregation and increased chain entanglement in anisole solution.^[^ ^97^ ^]^ Despite the film crystallinity was relatively low, well‐connected localized aggregates formed via short‐range π–π stacking and high tie‐chain density could play a crucial role in facilitating interchain charge hopping,^[^ ^104^, ^105^ ^]^ which might contribute to its high hole mobility and low activation energy.

Tapping‐mode atomic force microscopic (AFM) characterization was performed to probe the surface morphologies of those polymer thin films (Figure 8b). Smooth and featureless surface morphology was present in the annealed thin film of PTQBT and PTQBT‐V with small surface roughness in the range of 0.57−0.65 nm, while PTQBT‐2T exhibited a grain‐like morphology with a slightly higher surface roughness of 0.92 nm. In contrast, well‐defined and interconnected grains were observed in the thin film of PTQBT‐T, the surface roughness of which was further increased to 1.30 nm and agreed well with its highest crystallinity and ordered microstructure. In the case of the anisole processed PTQBT‐T thin film, it displayed a uniform surface with distinguishable grains, which could support efficient charge carrier transport. The surface roughness was decreased to 0.85 nm because of its lowered crystallinity.

## 3 Conclusion

This study provides an innovative and potent design guideline for green solvent‐processable semiconducting polymers and marks a significant advance in the performance of organic electronic devices by green processing. Through covalently integrating donor, quinoid and acceptor units, a hybrid building block, which is structurally distinct from known electroactive units, is readily accessed through a three‐step synthesis. The three‐in‐one non‐symmetric structural unit showcases remarkably large and tunable molecular dipole moment as well as a highly planar geometry. Its unique electronic properties endow the resulting conjugated polymer PTQBT‐T with high polarity, non‐symmetric sequence, and rigid and coplanar backbone conformation. PTQBT‐T is readily dissolved in anisole, a green solvent, and shows strong interchain aggregation in solution that leads to localized aggregates upon solidification. Remarkably, PTQBT‐T‐based OFET devices processed from anisole achieve reliably high hole mobilities up to 2.30 cm^2^ V^‐1^ s^‐1^, one of the highest values for green solvent‐processed semiconducting polymers. Additionally, these devices exhibit commendable operational and storage stability, parameters that are essential for practical use. This study elaborates the unique structural and optoelectronic properties of the TQBT hybrid building block and its polymers, validating its effectiveness and potential in producing high performance semiconducting polymers that can be processed from green solvent. The strategic use of the hybrid approach, applicable to different donor‐acceptor combinations, paves the way to an infinite range of high‐performance semiconducting polymers. This advancement holds promises for the future of organic electronic devices, facilitating large‐scale manufacturing and commercialization through sustainable processes. The realization of green synthetic protocols for semiconducting polymers based on this hybrid approach is also imperative and essential, which remains to be explored and encourages future endeavors.

## Conflict of Interest

The authors declare no conflict of interest.

## Supporting information

Supporting Information

## Data Availability

The data that support the findings of this study are available from the corresponding author upon reasonable request.
