# Supplementary material for: A Three‐in‐One Hybrid Strategy for High‐Performance Semiconducting Polymers Processed from Anisole
Source: Adv Sci (Weinh). 2024 Apr 22;11(25):2401345. doi: 10.1002/advs.202401345 (PMC11220690; doi:10.1002/advs.202401345)
Supplement: Supplementary file 1 — Supporting Information [file ADVS-11-2401345-s001.pdf]

ADVANCED SCIENCE
Open Access

Supporting Information

for *Adv. Sci.*, DOI 10.1002/advs.202401345

A Three-in-One Hybrid Strategy for High-Performance Semiconducting Polymers Processed from Anisole

Cheng Liu, Huanhuan Liang, Runze Xie, Quanfeng Zhou, Miao Qi, Chongqing Yang, Xiaodan Gu, Yunfei Wang, Guoxiang Zhang, Jinlun Li, Xiu Gong, Junwu Chen, Lianjie Zhang, Zesheng Zhang, Xiang Ge, Yuanyu Wang, Chen Yang, Yi Liu and Xuncheng Liu**

Supporting Information

A Three-in-One Hybrid Strategy for High-Performance Semiconducting Polymers Processed from Anisole

Cheng Liu, Huanhuan Liang, Runze Xie, Miao Qi, Chongqing Yang, Xiaodan Gu, Yunfei Wang, Quanfeng Zhou, Guoxiang Zhang, Jinlun Li, Xiu Gong, Junwu Chen, Lianjie Zhang, Zesheng Zhang, Xiang Ge, Yuanyu Wang, Chen Yang, Yi Liu and Xuncheng Liu**

Corresponding Authors:

*xcliu3@gzu.edu.cn,

*yliu@lbl.gov

1. Basic Information of Anisole. (According to CHEM21,[1] Sanofi[2] and GlaxoSmithKline[3] solvent guide of common solvents)

Full Name: anisole; methyl phenyl ether; methoxybenzene

CAS Number: 100-66-3

Appearance: colorless

Chemical Formula: C_7H_8O 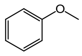

Molecular Weight: 108.14 g/mol

Density: 0.995 g/cm^3

Melting Point: –38°C

Boiling Points: 154°C

Flash Points: 52°C

Safety: 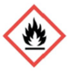 GHS02

ICH Limit: 5000 ppm

Occ. Health: OEBV2

Environment: EHB2

Worst H3xx: None

H4xx: None

Ranking after discussion: Recommended

Category: flavor and fragrance agents

2. General methods

Reagents were purchased from Energy Chemical, Innochem, Acros or synthesized as described. Dry solvents were collected from a solvent purification system. Proton and carbon nuclear magnetic resonance spectra (^{1}H-NMR and ^{13}C-NMR) spectra were recorded on a JNM-ECZ400S/L1 spectrometer. All chemical shifts are quoted using the δ scale, and all coupling constants (J) are expressed in Hertz (Hz). Cyclic voltammetry (CV) was carried out on a CHI600E electrochemical workstation with platinum electrodes at a scan rate of 50 mV s^{-1} against an Ag/Ag^+ reference electrode with nitrogen-saturated solution of 0.1 M tetrabutylammonium hexafluorophosphate (Bu_4NPF_6) in acetonitrile (CH_3CN). Potentials were referenced to the ferrocenium/ferrocene couple by using ferrocene as an internal standard. The molecular weight of polymer was determined by high temperature size exclusion chromatography (SEC) at 140 °C using a calibration curve of polystyrene standards, with 1,2,4-trichlorobenezene as the eluent. UV-Vis-NIR spectra were recorded using a MAPADA UV-6300 spectrometer. GIWAXS[4] data were acquired at Beamline 7.3.3 of Advanced Light Source, Lawrence Berkeley National Laboratory, respectively. Atomic force microscopy (AFM) images were obtained with a Bruker Dimension ICON, using tapping mode in air under ambient conditions. The C–V measurements were conducted based on HP 4192A LCR meter by sweeping the voltage from –10 to 10 V at room temperature, with a ramping rate of 0.5 V s^{-1} and 30 mV of oscillator levels, where the frequency was from 1×10^3 to 1×10^6 Hz.

3. Synthesis procedures

(Z)-1-acetyl-3-((5-bromothiophen-2-yl)methylene)piperazine-2,5-dione (2)

A mixture of **1** (990.9 mg, 5 mmol, 1 eq.), 5-Bromothiophene-2-carbaldehyde (995.2 mg, 5 mmol, 1 eq), Cs_2CO_3 (714.7 mg, 5.25 mmol, 1.05 eq.) was dissolved in 50 mL of anhydrous DMF under N_2 protection. The reaction mixture was stirred at room temperature for 5h. Then

crushed ice was added and then yellow precipitate was formed, the precipitate was collected by filtration and rinsed with ethyl alcohol. Yield: 80%. ^{1}H NMR ($CDCl_3$, 400 MHz, 298 K) δ = 7.86 (s, 1H), 7.18 (d, *J* = 0.8 Hz, 1H), 7.13 (d, *J* = 4.0 Hz, 1H), 7.07 (dd, *J* = 3.9, 0.8 Hz, 1H), 4.52 (s, 2H), 2.62 (s, 3H). ^{13}C NMR ($CDCl_3$, 101 MHz, 298 K) δ = 172.40, 162.98, 160.22, 137.01, 131.44, 131.13, 123.89, 116.73, 112.73, 46.05, 27.21.

(3Z,6Z)-3-((7-bromobenzo[*c*][1,2,5]thiadiazol-4-yl)methylene)-6-((5-bromothiophen-2-yl)methylene)piperazine-2,5-dione (3)

Into a mixture of **2** (2633.3 mg 8.0 mmol, 1 eq.) and 7-bromobenzo[*c*][1,2,5]thiadiazole-4-carbaldehyde (1944.6 mg, 8 mmol, 1 eq.) in DMF (50 mL) was syringe injected triethylamine (50 mL) at 130 °C under N_2 protection. Yellow precipitate was formed during the overnight reaction. After cooling to room temperature, the precipitate was collected by filtration and rinsed with ethyl acetate. The resulting yellow solid was vacuum dried. The product was used for next steps without further purification. Yield: 86%.

TQBT-2Br-C6 (4)

A mixture of the compound **3** (1024.3 mg, 2mmol, 1 eq.), K_2CO_3 (1105.6 mg, 8 mmol, 4 eq.) and 1-bromohexane (1320.5 mg, 8 mmol, 4 eq.) in DMF (60 mL) was stirred at 130 °C for 6 hours under N_2 protection. After cooling to room temperature, the reaction mixture was filtered, and the filtrate was concentrated by rotary evaporation under reduced pressure. And the crude product was subjected to column chromatography (Hexane: $CHCl_3$ (5:1 [v/v]) and then was recrystallized twice to afford the desired product as bright red solid. Yield: 30%.

^{1}H NMR ($CDCl_3$, 400 MHz, 298 K) δ = 8.94 (d, *J* = 8.0 Hz, 1H), 7.89 (d, *J* = 7.9 Hz, 1H), 7.83 (s, 1H), 7.13 (s, 1H), 7.04 (s, 2H), 4.55 (t, *J* = 6.9 Hz, 2H), 4.42 (t, *J* = 6.6 Hz, 2H), 1.99 (d, *J* = 7.6 Hz, 1H), 1.88 (d, *J* = 7.7 Hz, 1H), 1.44 – 1.22 (m, 14H), 0.99 – 0.86 (m, 6H). ^{13}C NMR ($CDCl_3$, 101 MHz, 298 K) δ = 159.03, 158.09, 157.13, 154.78, 140.42, 133.49, 132.94, 130.88, 130.31, 129.51, 129.01, 128.04, 119.73, 117.99, 114.89, 112.71, 68.64, 67.32, 31.72, 28.70, 28.59, 26.09, 22.83, 22.75, 14.22.

TQBT-2Br-DT (5)

A mixture of the compound **3** (1024.3 mg, 2mmol, 1 eq.), K_2CO_3 (1105.6 mg, 8 mmol, 4 eq.) and 11-(bromomethyl)tricosane (3340.4 mg, 8 mmol, 4 eq.) in DMF (70 mL) was stirred at 130 °C for 6 hours under N_2 protection. After cooling to room temperature, the reaction mixture was filtered, and the filtrate was concentrated by rotary evaporation under reduced pressure. And the crude product was subjected to column chromatography (Hexane: $CHCl_3$ (6:1 [v/v]) and then was recrystallized twice to afford the desired product as bright red solid. Yield: 28%.

^{1}H NMR ($CDCl_3$, 400 MHz, 298 K) δ = 8.95 (d, *J* = 8.0 Hz, 1H), 7.88 (d, *J* = 8.0 Hz, 1H), 7.85

(s, 1H), 7.09 (s, 1H), 7.04 (s, 2H), 4.42 (d, J = 6.1 Hz, 2H), 4.32 (d, J = 5.6 Hz, 2H), 2.09 – 1.95 (m, 1H), 1.94 – 1.84 (m, 1H), 1.42 – 1.15 (m, 80H), 0.85 (td, J = 6.1, 5.4, 1.9 Hz, 12H). ^{13}C NMR ($CDCl_3$, 101 MHz, 298 K) δ = 159.17, 158.32, 154.82, 153.17, 140.44, 133.46, 132.92, 130.81, 130.27, 129.42, 129.04, 128.17, 119.76, 117.91, 115.00, 112.69, 77.33, 71.74, 70.00, 37.44, 37.26, 32.03, 31.89, 31.73, 30.27, 30.16, 29.87, 29.81, 29.78, 29.48, 27.01, 26.97, 22.81, 14.26.

General procedure for Stille polymerization (with polymer PTQBT-T as an example).
A mixture of monomer **5** (355.64 mg, 0.30 mmol, 1 eq.), 2,5-bis(trimethylstannyl) thiophene (122.91 mg, 0.3 mmol, 1 eq.), $Pd(PPh_3)_4$ (10.38 mg, 9 μmol, 0.03 eq.) in toluene (12 mL) was sealed in a Ar flushed vessel and heated to 120 °C while stirring for 24 h. After cooling down, the mixture was precipitated into methanol. The precipitate collected from filtration was subjected to Soxhlet extraction with methanol, acetone, ethyl acetate, and chloroform (CF) successively. The CF fraction was concentrated and precipitated into methanol, respectively. The polymer PTQBT-T was collected by filtration. Mn=17,822, Mw=45,955, PDI =2.57.

PTQBT: Mn=14,581, Mw=27,330, PDI =1.87.

PTQBT-V: Mn=13,730, Mw=331503, PDI =2.29.

PTQBT-2T: Mn=15,571, Mw=29,330, PDI =1.88.

4. Fabrication and characterization of field effect transistors (OFETs)

Polymer thin film field effect transistors were fabricated in a typical bottom gate, top contact architecture. Transistors were fabricated with highly doped Si as the gated electrode, gold (Au) as both source and drain electrodes. Substrates were cleaned by successive sonication with soap water, deionized water, acetone and absolute ethanol. Then the substrate gate dielectric layers were modified by *n*-octadecyltrichlorosilane (OTS) by submersion in a solution of OTS in toluene. The four polymer films for comparison were all prepared from pplymer solution (CB, 5.0 mg/mL) by spin-coating (3000 rpm, 30 s) onto the OTS treated substrates. Besides, PTQBT-T in anisole (2 mg/ml) was spin-coated (2000 rpm, 35 s) onto the OTS treated substrates to form polymer thin films. When thermal treatment was noted, the polymer films were annealed at 200 °C or 150°C for 15 minutes on a hotplate in a nitrogen glovebox. Gold contacts (40 nm) were evaporated on the polymer film layer through a metal mask to define channels of 80 μm in length and 1400 μm in width. The film thickness of the devices ranges from 60 to 100 nm. Field effect mobility was calculated from the standard equation for saturation region in metal-dioxide-semiconductor field effect transistors: $I_{ds} = \mu\ (W/2L)\ C_i\ (V_g - V_t)^2$, where I_{ds} is drain-source current, μ is field effect mobility, W and L are the channel width and length, C_i is the

capacitance per unit area of the gate insulator (C_i = 10 nF/cm^2), V_g is the gate voltage and V_t is the threshold voltage.

5. Theoretical calculations

For simplicity, the alkyl chains were all replaced by methyl groups. Density functional theory (DFT) calculations of building block, monomer and trimer of polymers were performed using Gaussian 09[5] at the B3LYP[6]/6-311G (d, p)[7] level with the D3 (BJ) empirical dispersion correction.[8] The calculation of band structures and density of states of the polymers were performed using Vienna ab initio simulation package (VASP)[9] with the Perdew-Burke-Ernzerhof (PBE) functional instead of B3LYP functional due to its mild demands of computational resources and the importance of being consistent with previous related studies.[10] Uniform 21 × 1 × 1 Monkhorst-Pack *k*-point mesh was used for structural optimization. The energy cut off for the plane-wave expansion was set to 400 eV and the force criteria was less than 0.05 eV/Å. 41 *k*-points were calculated between the gamma point and the edge of the first BZ to afford band structure and density of states. The hole effective mass (m_h*)[11] for 1D crystal is calculated based on band structure by the equation:

$m_h^* = h^2 / (d^2E/dk^2)$

Where E is the band energy and k is the electron wave vector along backbone direction.

6. Solubility Limit Measurement

The solubility of PTQBT-based polymers were measured by a standard calibration curve method.[12] Firstly, to construct standard calibration curves, the absorbance of different concentrations of PTQBT, PTQBT-V, PTQBT-T and PTQBT-2T polymer (0.01−0.05 mg/mL) in CB and PTQBT-T polymer (0.01−0.07 mg/mL) in anisole solutions were measured. Then, the polymer was gradually added to the solution until saturation was achieved, the CB and anisole solution were stirred at 70°C for four hours. The solution was then cooled to room temperature and centrifuged (e.g., 10000 rpm for 30 min). Finally, the top clear solution in each centrifuge tube was selected, and the solubility of each material was determined by measuring its absorbance after dilution with a saturated solution and using individual standard curves.

7. Additional graphs

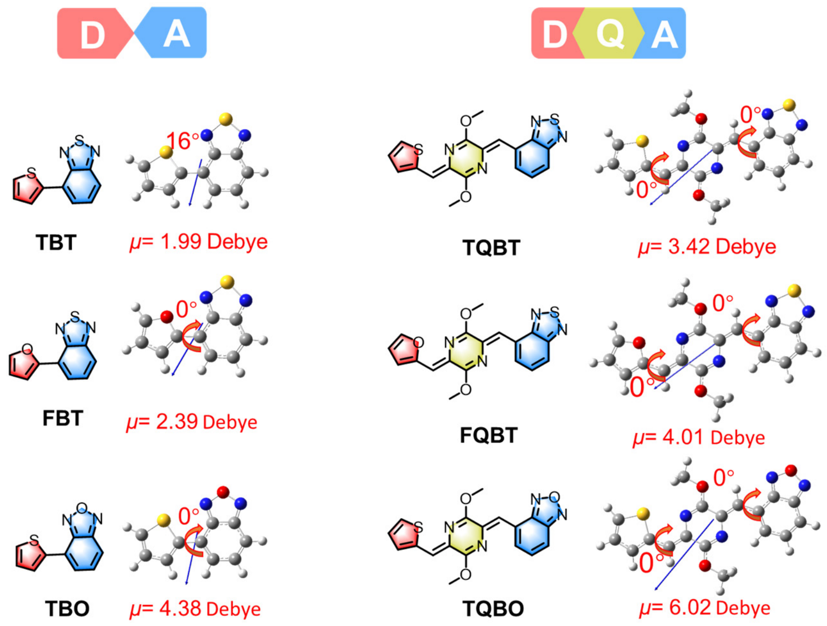

Figure S1. Calculated dipole memonts of D-A building block and their corresponding D-Q-A building block.

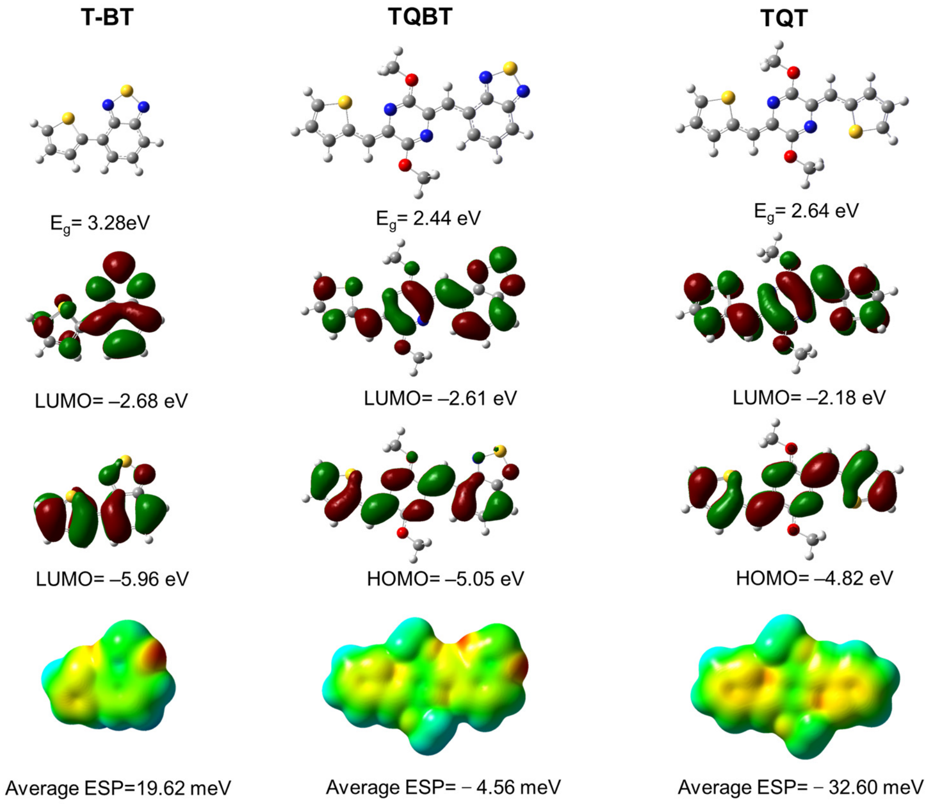

Figure S2. Calculated molecular orbital, energy level, bandgap and electrostatic potential (ESP) distributions of T-BT, TQBT and TQT unit.

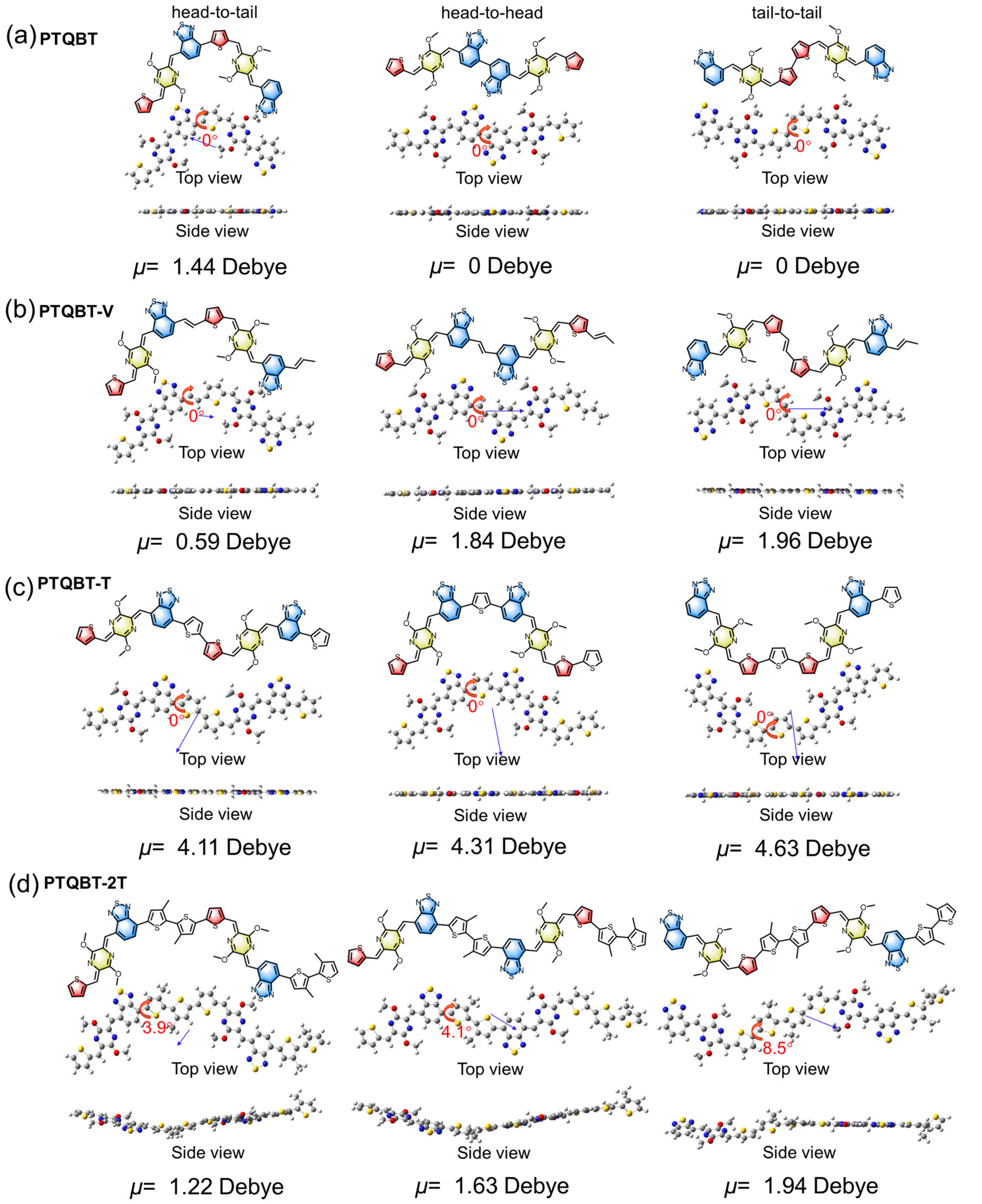

Figure S3. Optimized geometries and dipole moments for the three possible segments of the dimeric units of four TQBT-based polymers.

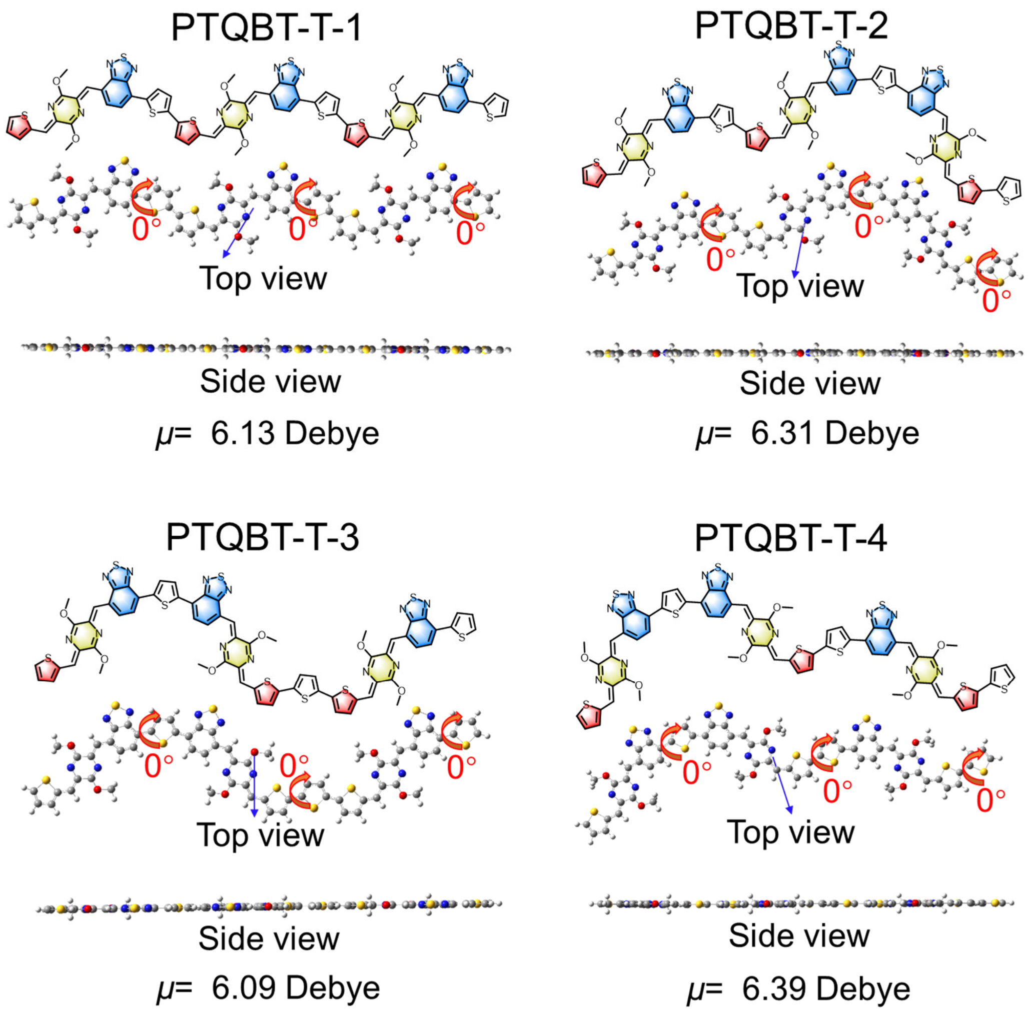

Figure S4. Optimized geometries and dipole moments for the four possible trimers with different TQBT orientations in the copolymer PTQBT-T.

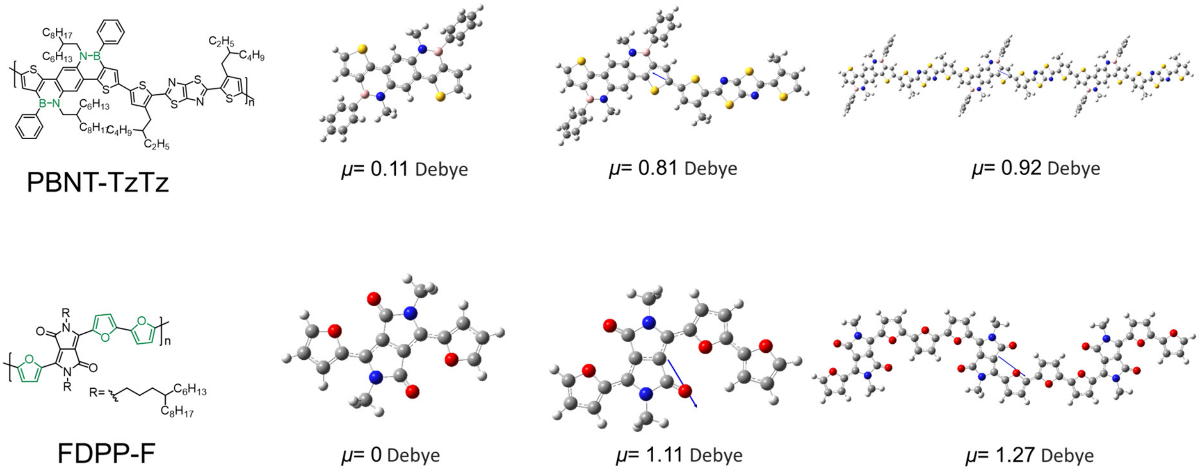

Figure S5. Calculated diople moments of the corresponding building block, monomer and trimer of previouly reported polymer PBNT-TzTz and FDPP-F.

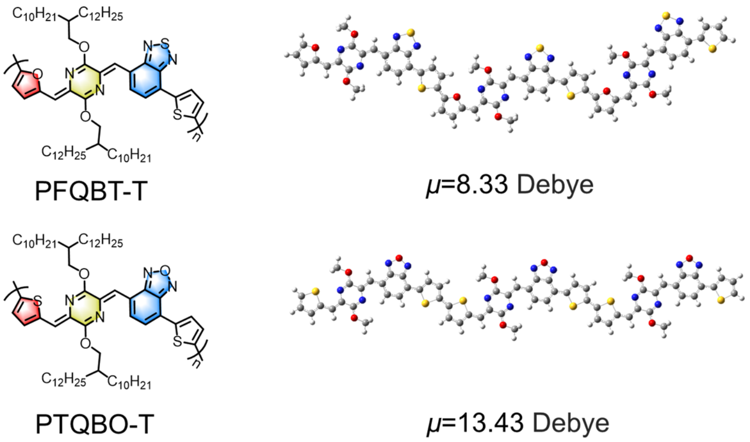

Figure S6. Calculated diople moments of the trimers of PFQBT-T and PTQBO-T.

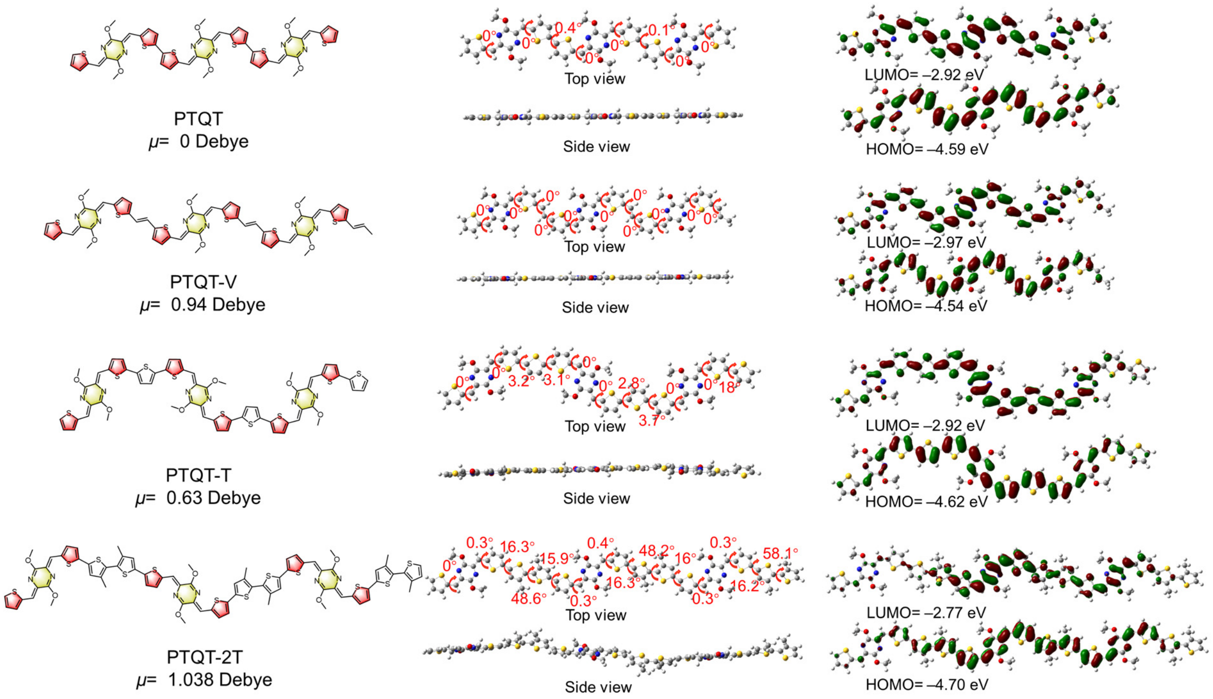

Figure S7. Calculated dipole moments, optimized geometries, orbital distributions and energy levels of the trimers of TQT-based polymers.

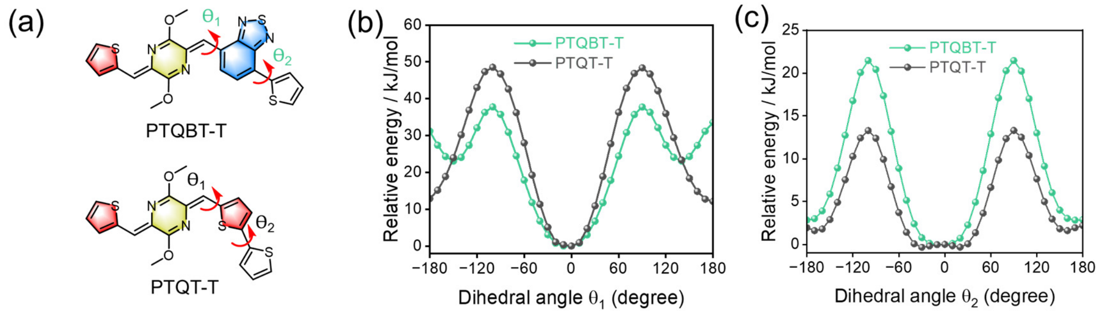

Figure S8. (a) Theoretical model for the calculation of torsional potential energy. Calculated torsional energy barrier as a function of b) dihedral angel (θ_1) and c) dihedral angel (θ_2).

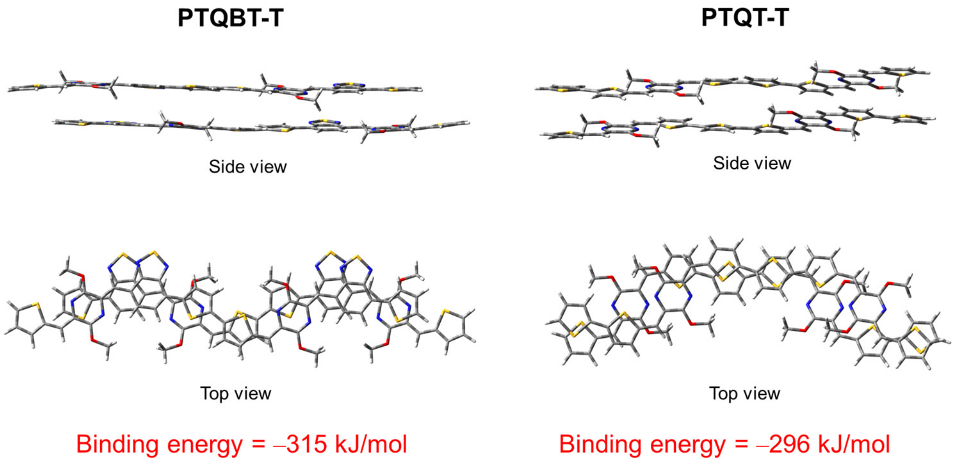

Figure S9. Optimized packing geometries and calculated binding energies of PTQBT-T and PTQT-T dimers.

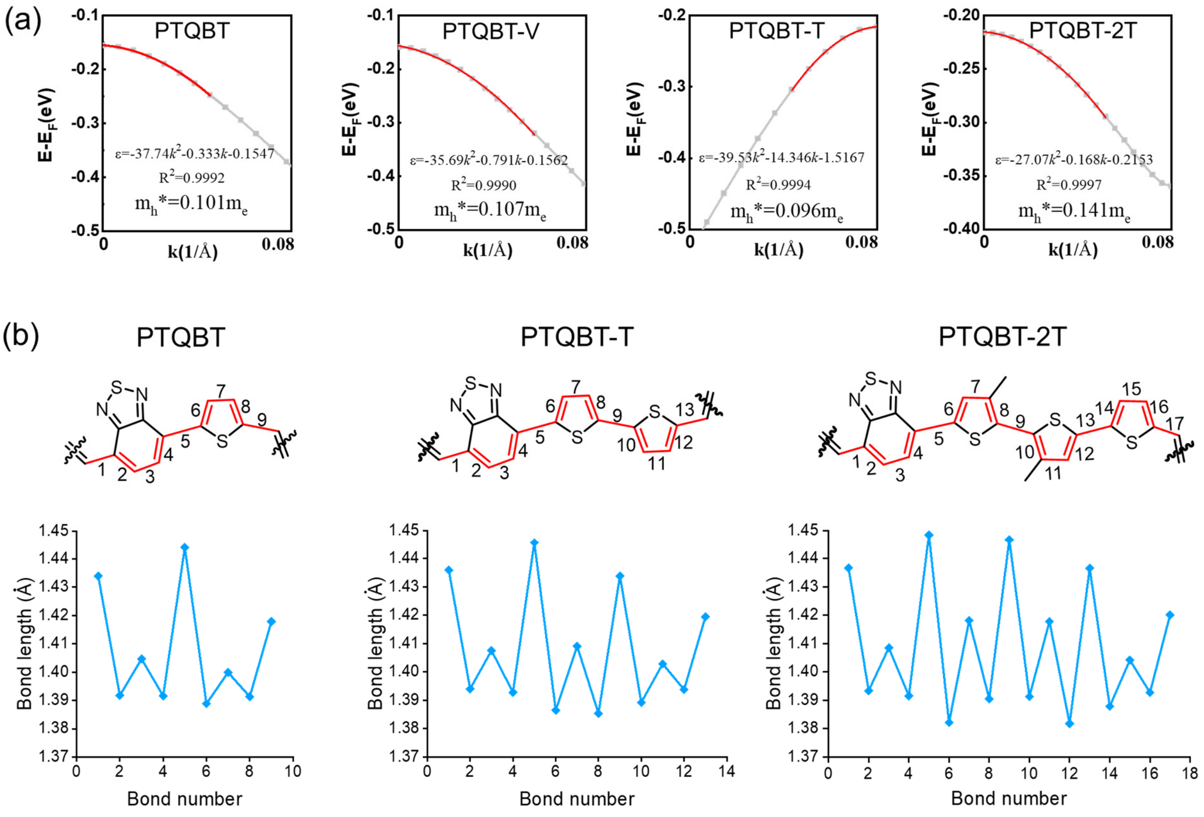

Figure S10. a) Curve fitting based on band structure to obtain the effective hole masses of four polymers. b) Plots of carbon−carbon bond length for each respective bond number in benzothiadiazole and oligothiophene segments in PTQBT, PTQBT-T and PTQBT-2T.

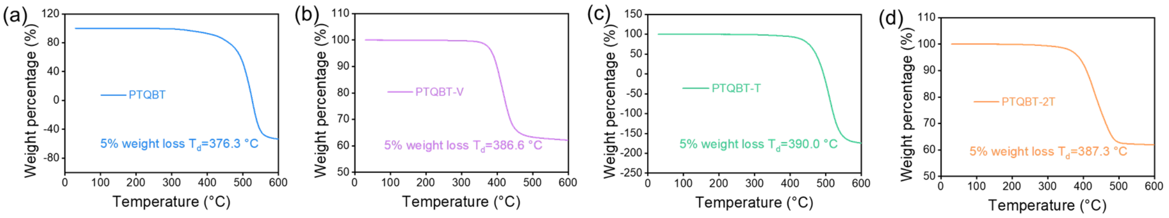

Figure S11. Thermogravimetric analysis (TGA) analysis curves of four polymers.

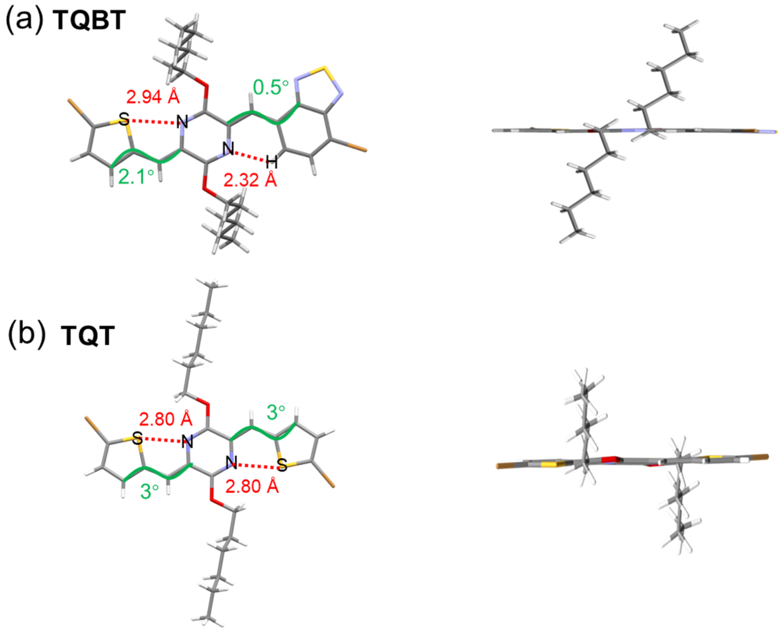

Figure S12. X-ray structures of a) TQBT and b) TQT building block.

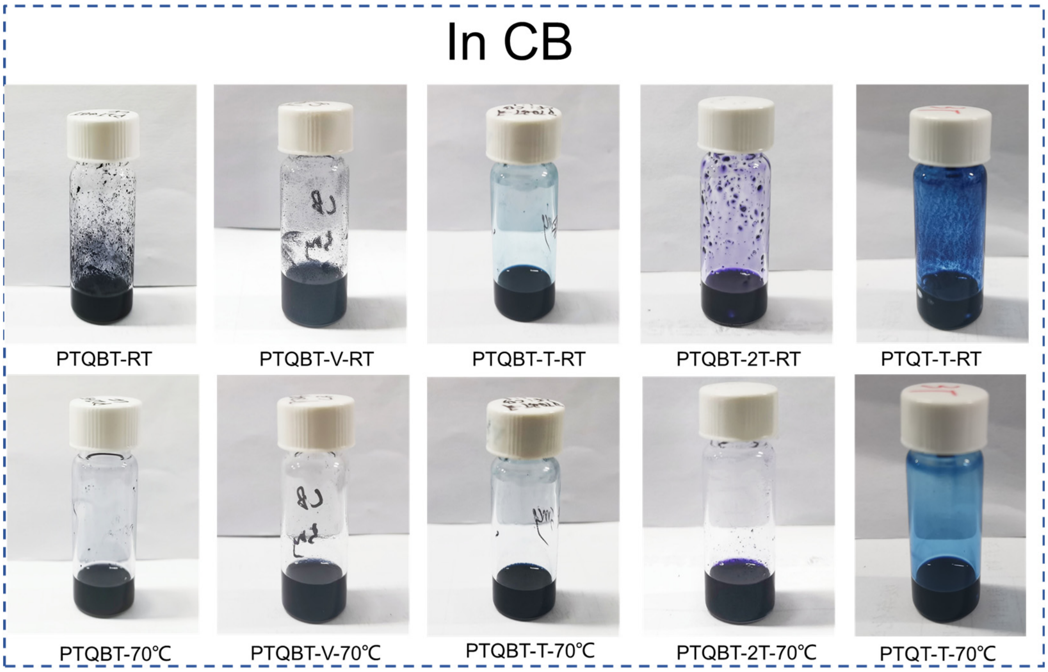

Figure S13. Photographs of five polymers in chlorobenzene.

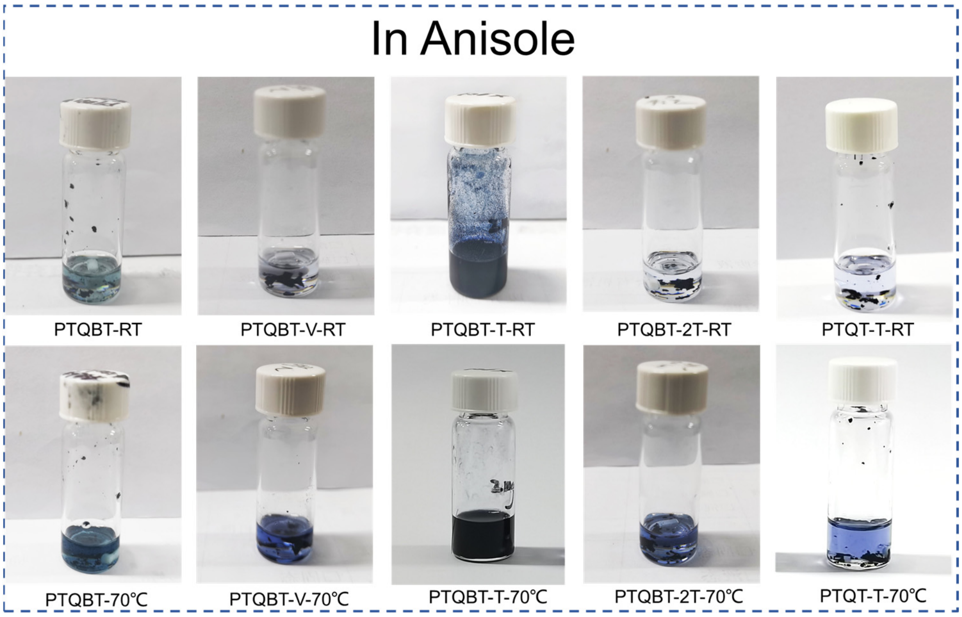

Figure S14. Photographs of five polymers in anisole.

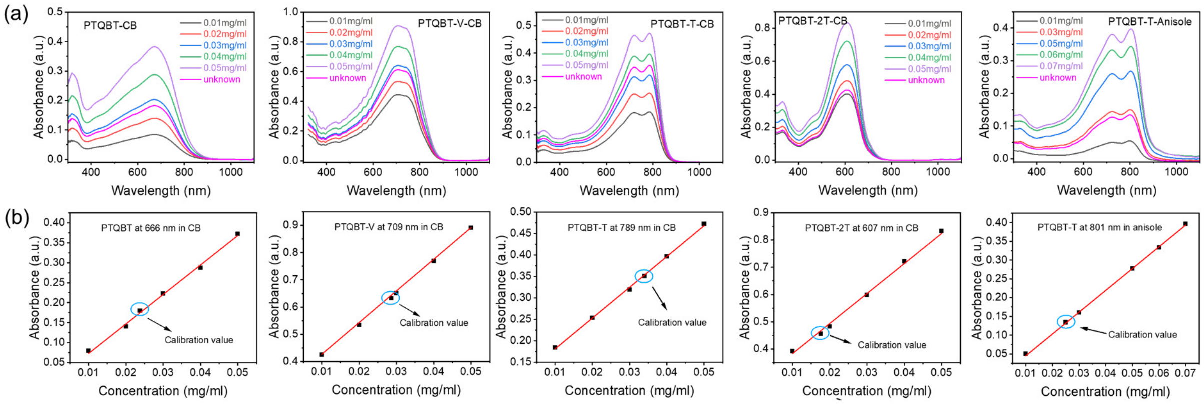

Figure S15. Absorption spectra for determining the solubility of four polymers in 70 °C chlorobenzene and anisole: a) absorption spectra of standard solution four polymers. b) plot of absorbance at a certain wavelength (as indicated in the figures) versus concentration. The unknown PTQBT, PTQBT-V, PTQBT-T, PTQBT-2T and PTQBT-T-Anisole based samples were prepared by dilution of 600, 600, 600, 600, 300 times from saturated solution.

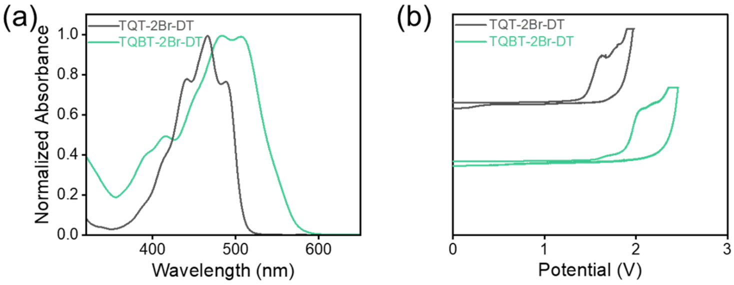

Figure S16. a) Normalized UV-vis absorption spectra of TQBT-2Br-DT and TQT-2Br-DT in chlorobenzene. b) Cyclic voltammetry curves of TQBT-2Br-DT and TQT-2Br-DT at a scan rate of 100 mV/s.

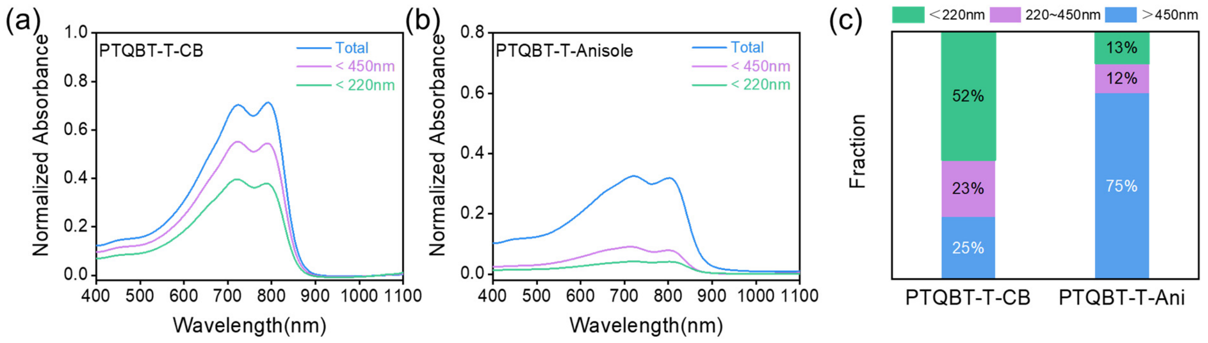

Figure S17. Absorption spectra of PTQBT-T solution in a) chlorobenzene and b) anisole in the filtration experiments.[13] c) Size distributions of solution aggregates of PTQBT-T in chlorobenzene and anisole at room temperature.

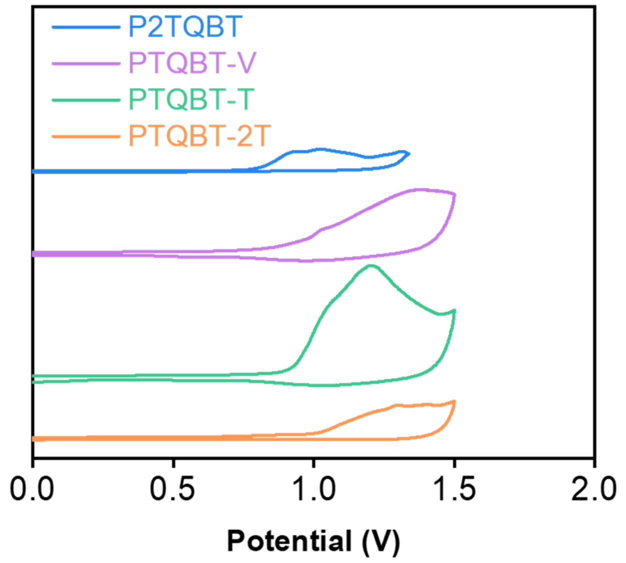

Figure S18. Cyclic voltammetry curves of polymers at a scan rate of 100 mV/s.

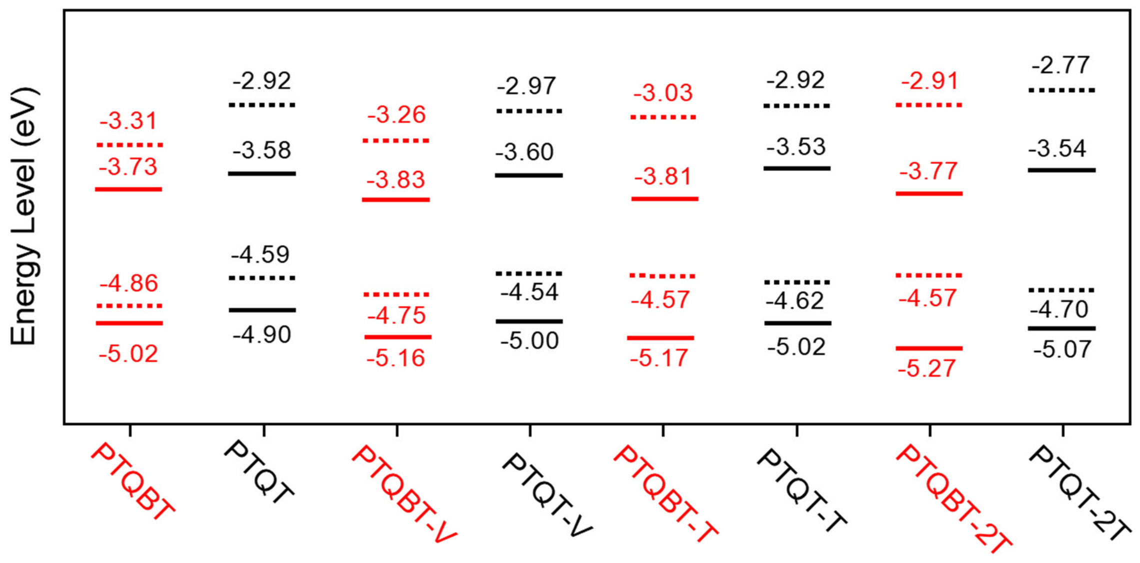

Figure S19. Calculations (dash) and experimental (solid) HOMO and LUMO levels of TQBT-based polymers (red) and TQT-based polymers (black).

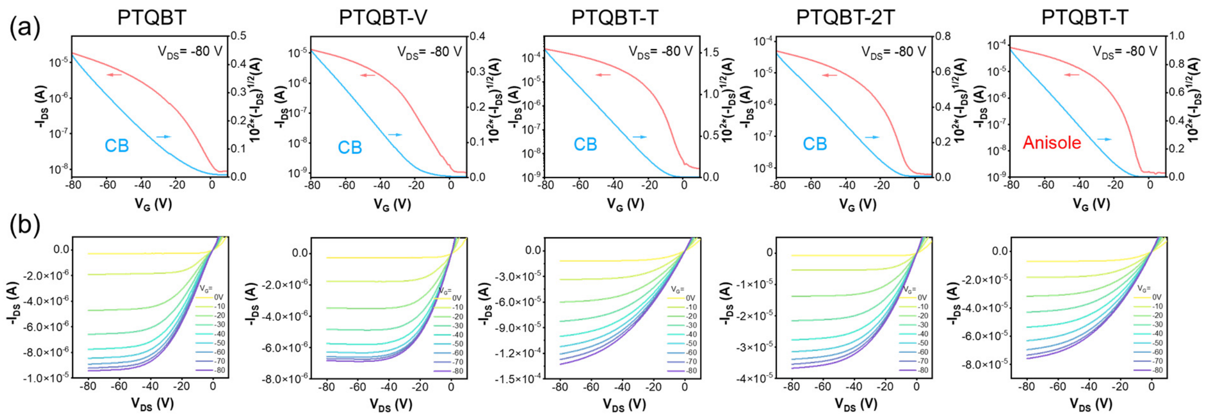

Figure S20. Typical a) transfer and b) output characteristics of OFETs without thermal annealing.

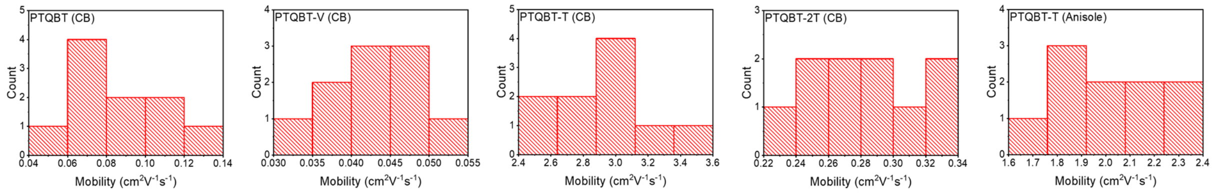

Figure S21. The mobility statistic graphs for OFETs based on annealed TQBT-based polymer films.

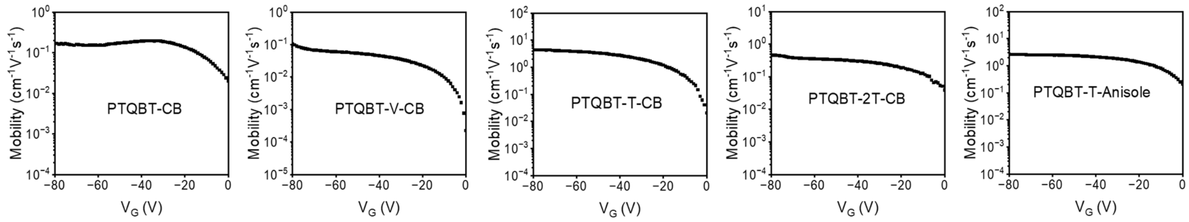

Figure S22. Mobility *versus* gate voltage plots of annealed OFETs based on four TQBT-based polymers spuncast from chlorobenzene or anisole.

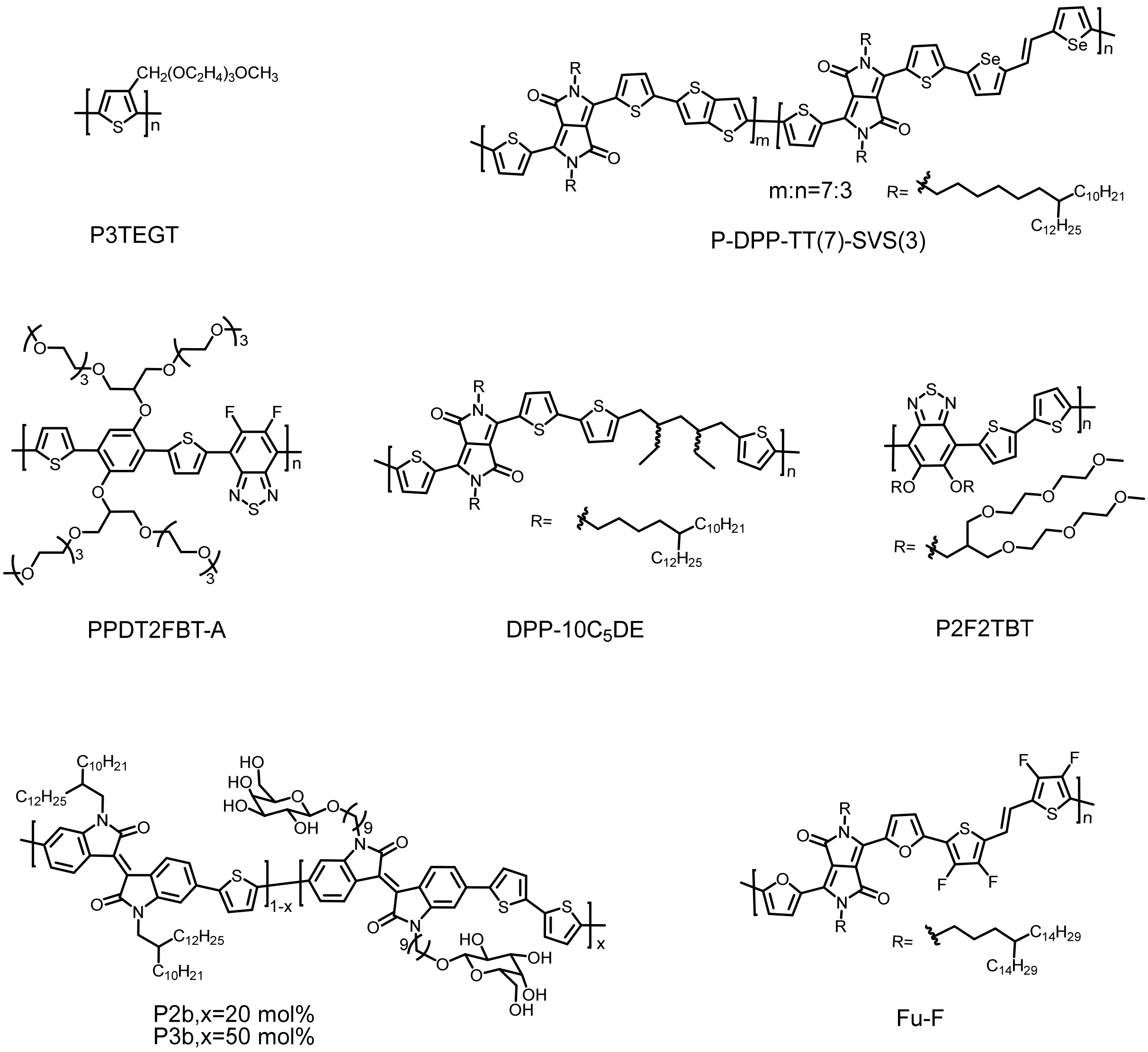

Figure S23. Chemical structures from the literature of green solvent-processed polymers by spin-coating deposition method in recent 10 years.

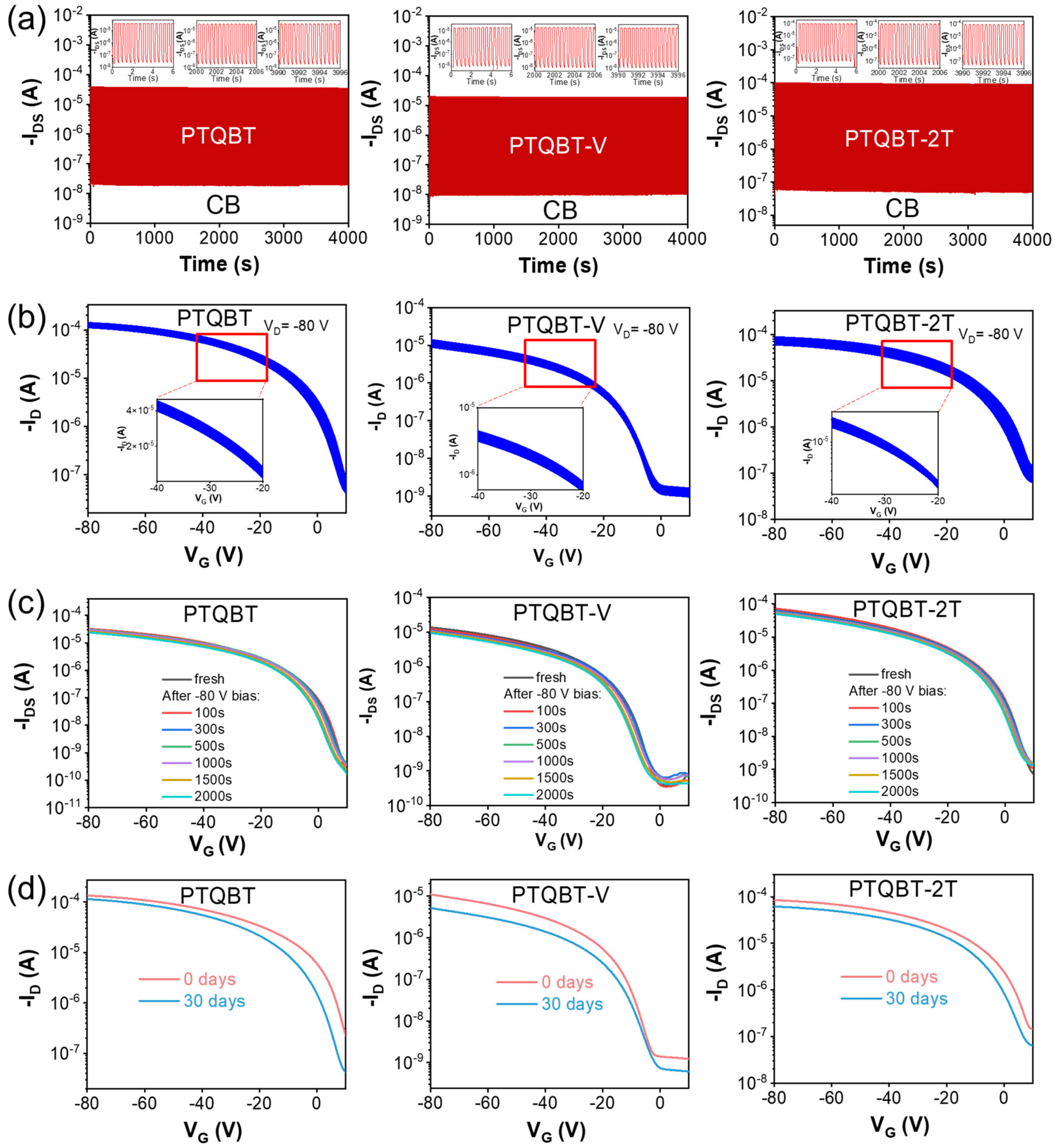

Figure S24 a) On-off cyclic tests (9000 cycles) by applying V_G of −80 and 0 V at V_D of −80 V, b) Repeatability of transfer curves (30 cycles) by applying V_D of −80 V, c) Bias stress tests by applying continuous bias voltage of −80 V for up to 2000 s and d) Transfer curves before and after storing in ambient air for 30 days of OFET devices based on PTQBT, PTQBT-V and PTQBT-2T.

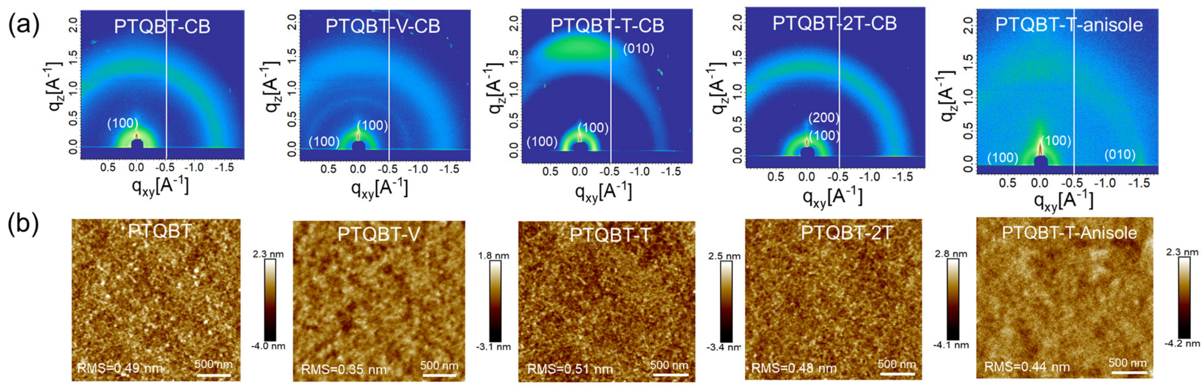

Figure S25. a) GIWAXS patterns and b) AFM images of as-cast polymer films.

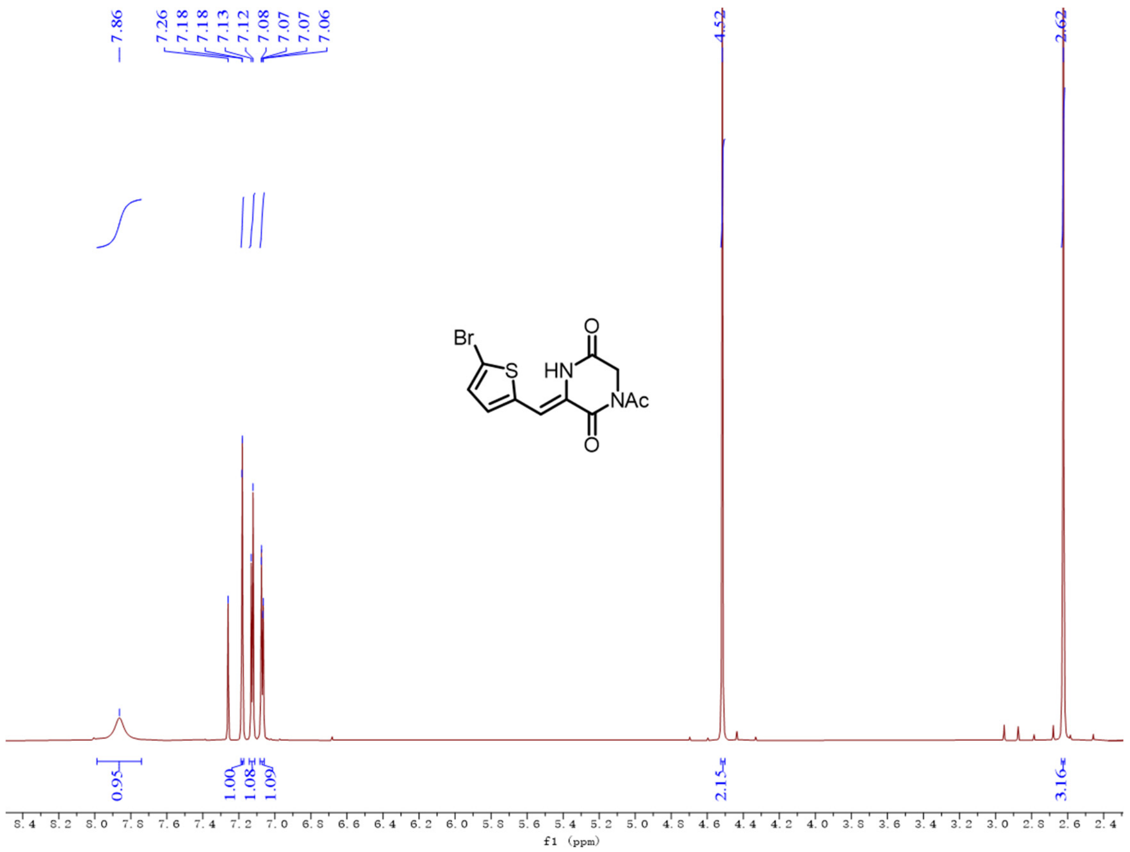

Figure S26. ^{1}H NMR spectrum of compound 2 ($CDCl_3$, 298 K).

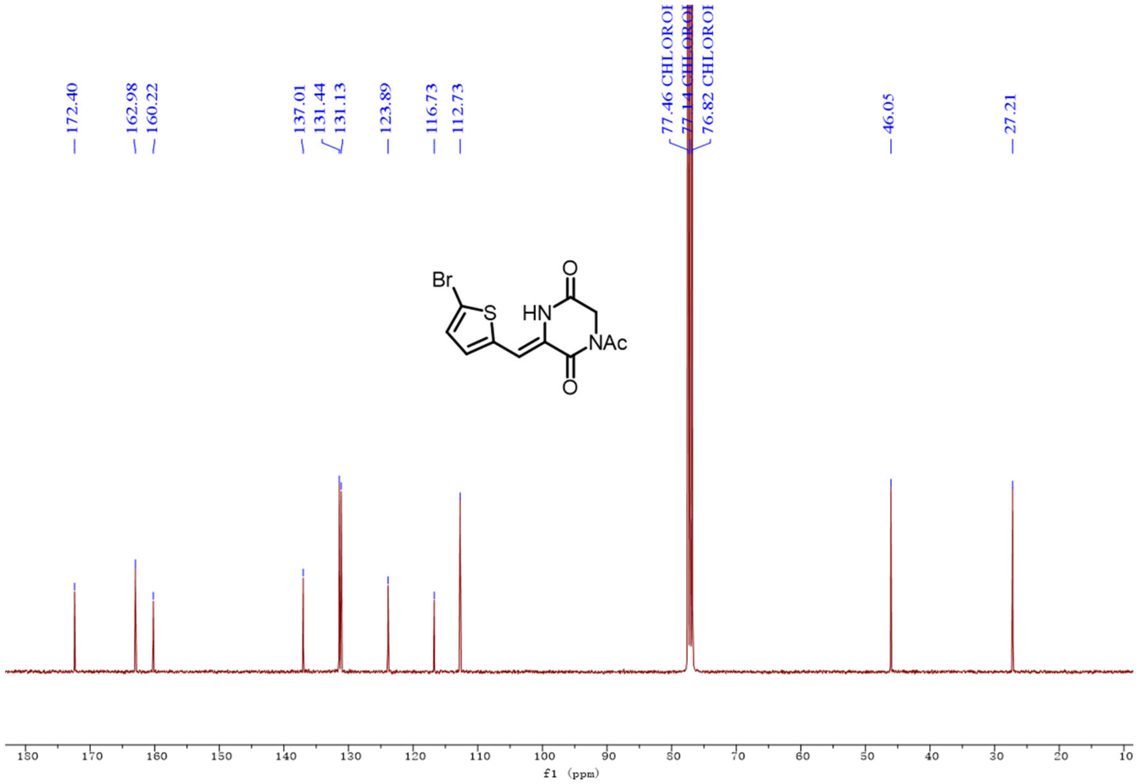

Figure S27. ^{13}C NMR spectrum of compound 2 ($CDCl_3$, 298 K).

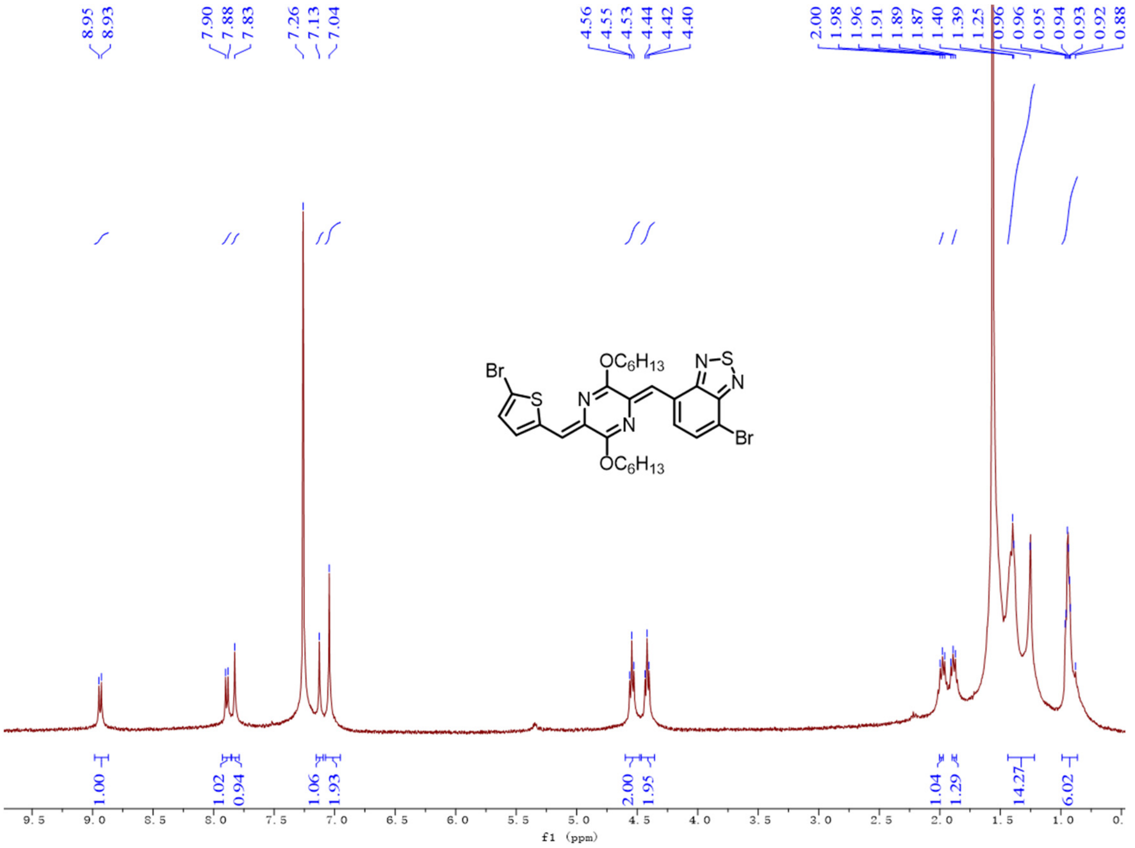

Figure S28. ^{1}H NMR spectrum of monomer 4 ($CDCl_3$, 298 K).

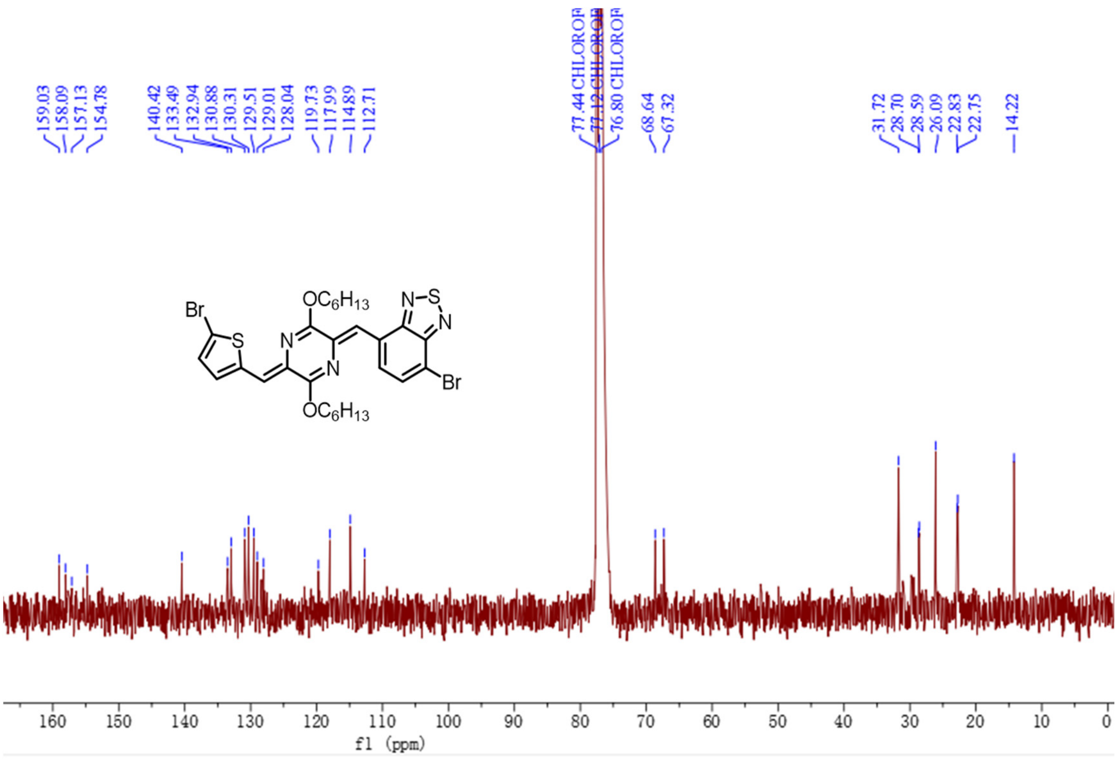

Figure S29. ^{13}C NMR spectrum of monomer 4 ($CDCl_3$, 298 K).

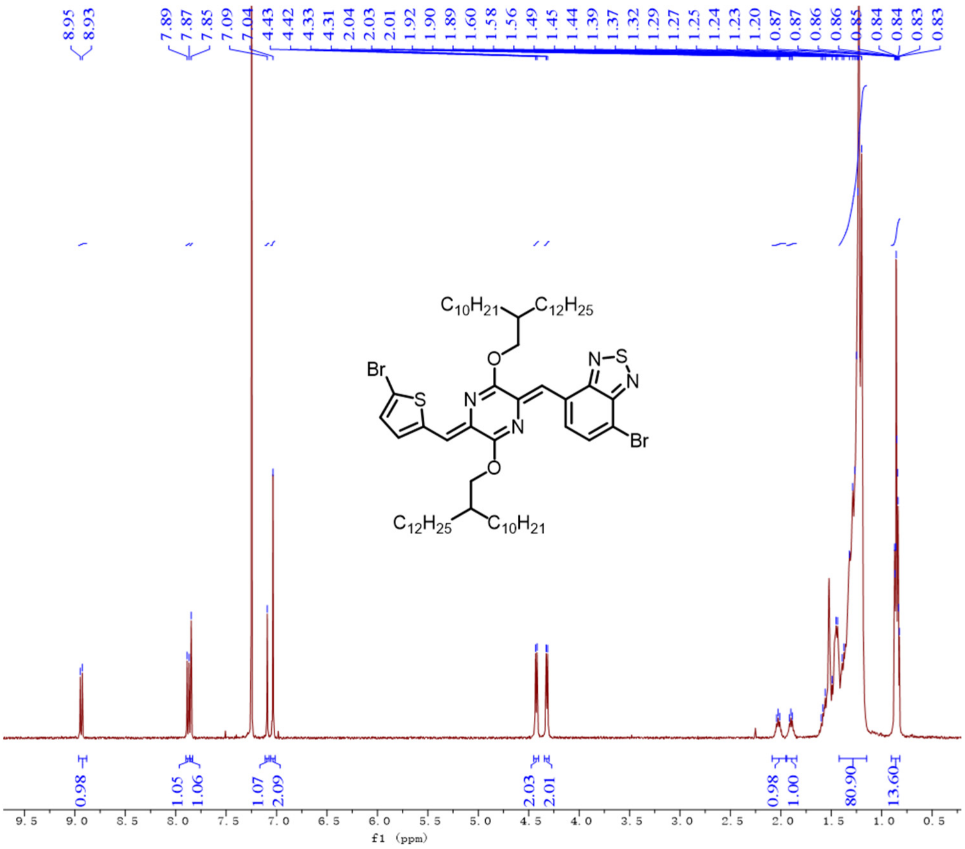

Figure S30. ^{1}H NMR spectrum of monomer 5 ($CDCl_3$, 298 K).

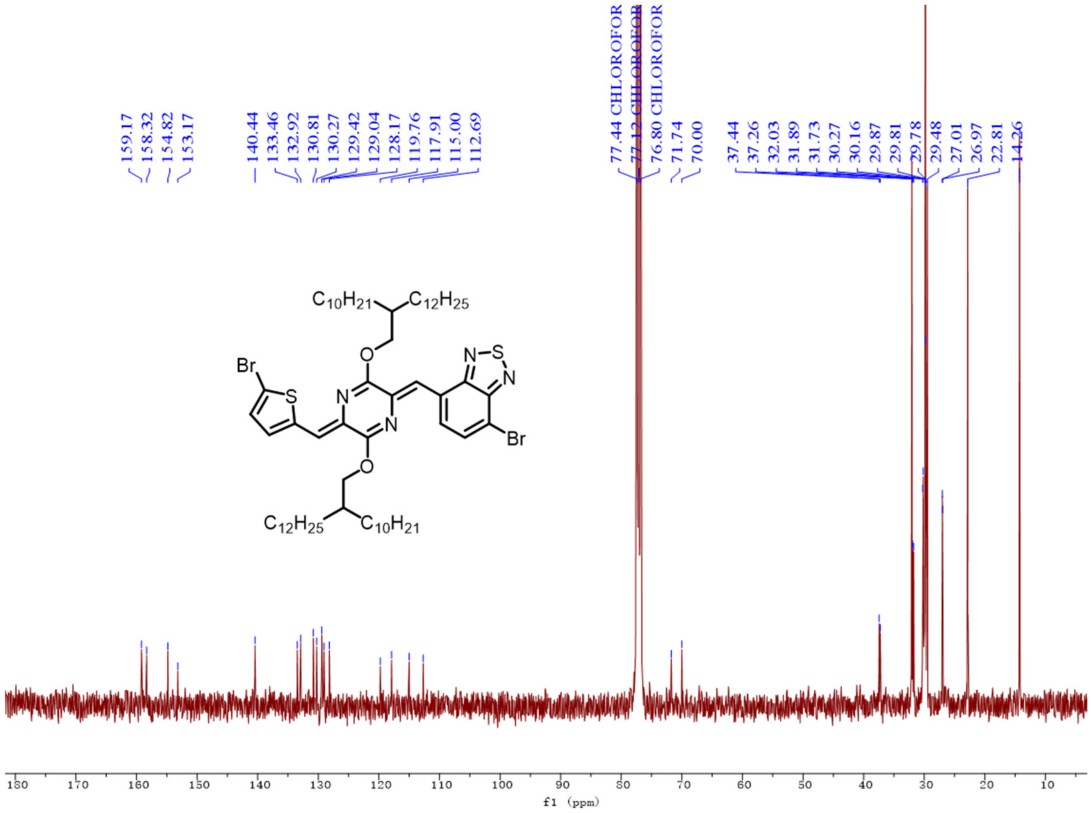

Figure S31. ^{13}C NMR spectrum of monomer 5 ($CDCl_3$, 298 K).

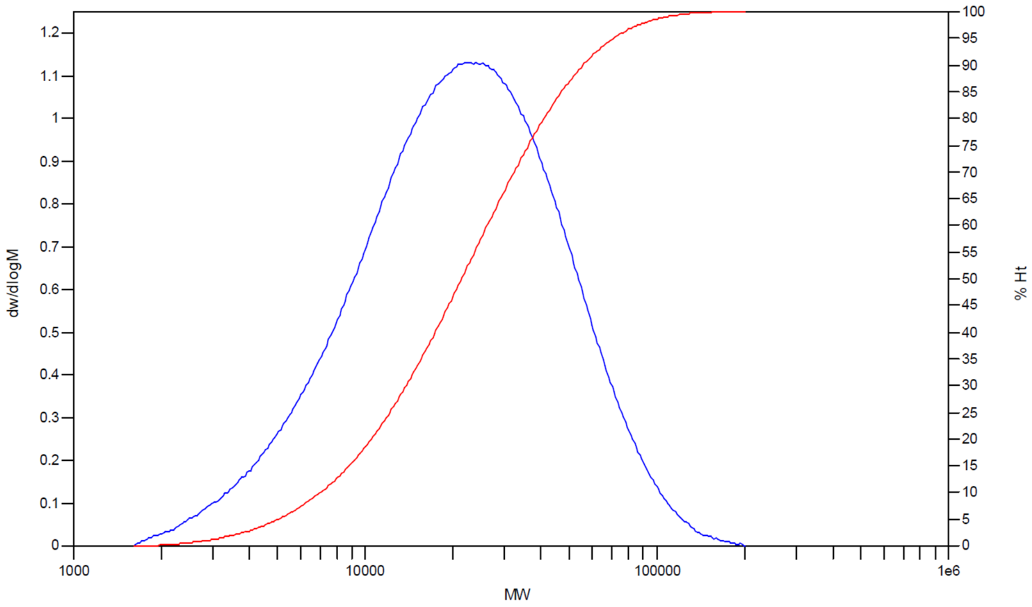

Figure S32. SEC distribution plots of PTQBT with 1,2,4-trichlorobenezene as the eluent at 140 °C.

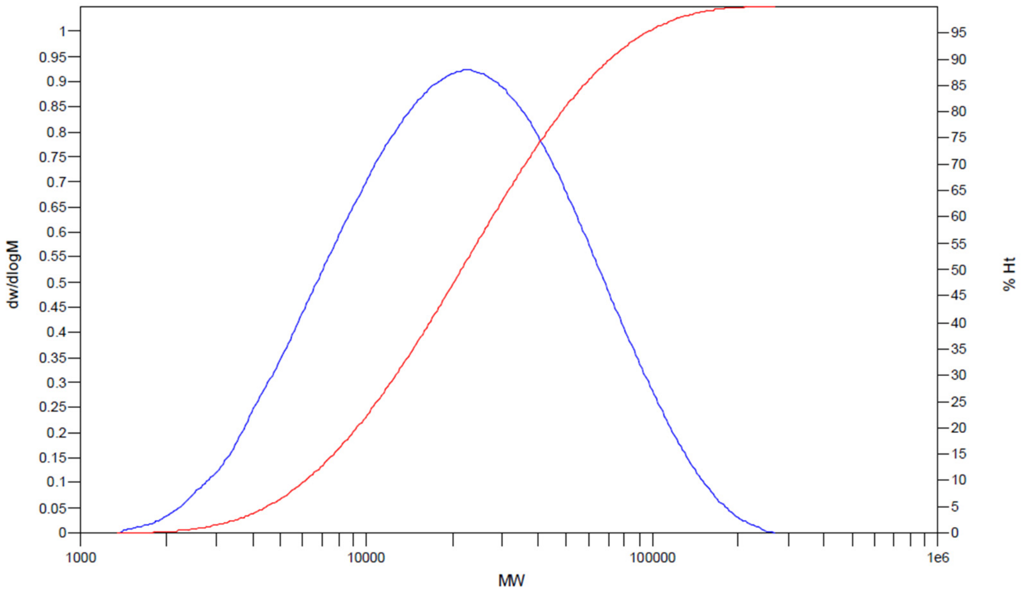

Figure S33. SEC distribution plots of PTQBT-V with 1,2,4-trichlorobenezene as the eluent at 140 °C.

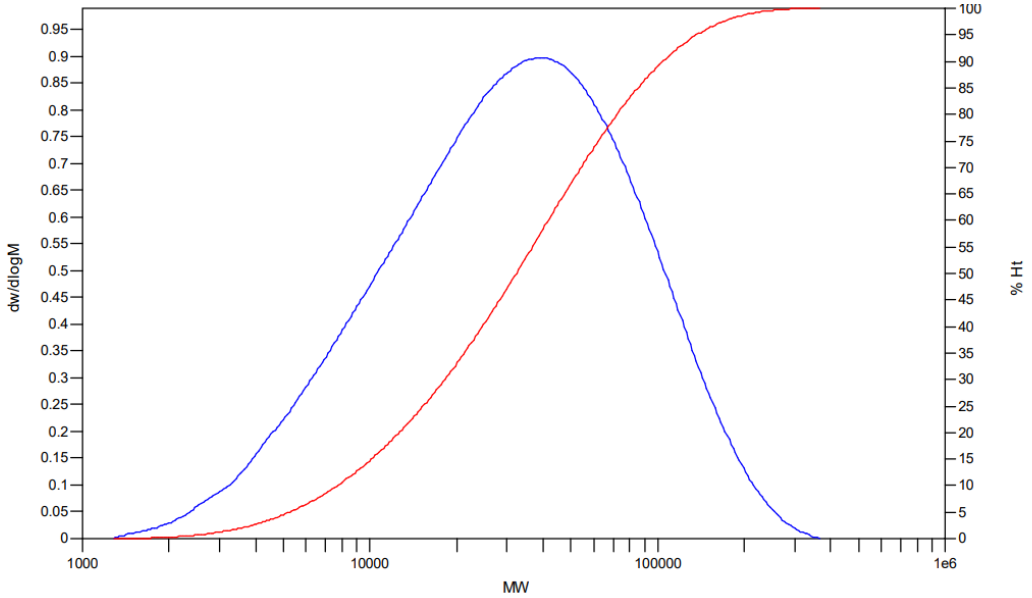

Figure S34. SEC distribution plots of PTQBT-T with 1,2,4-trichlorobenezene as the eluent at 140 °C.

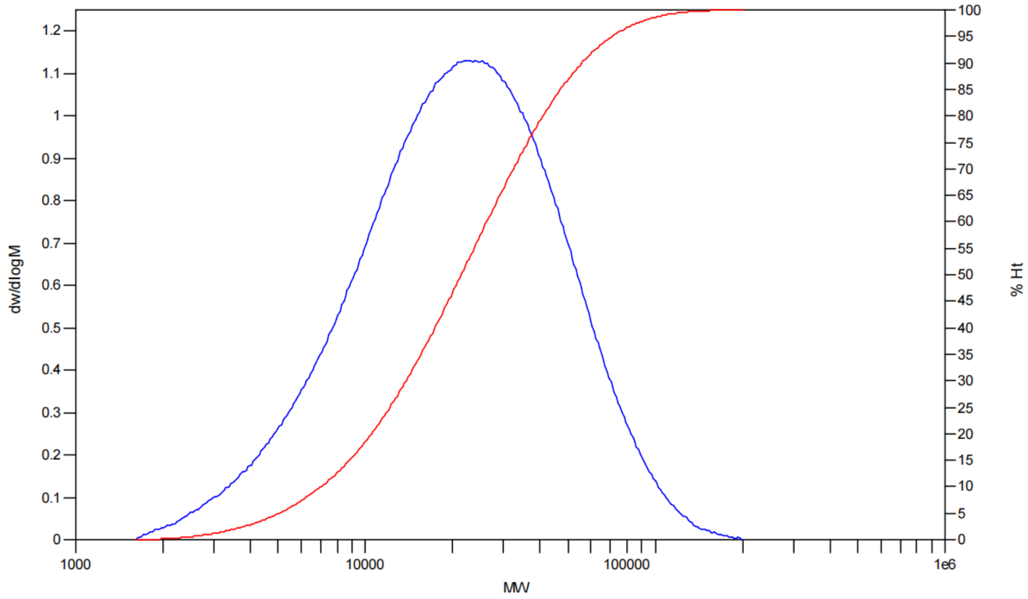

Figure S35. SEC distribution plots of PTQBT-2T with 1,2,4-trichlorobenezene as the eluent at 140 °C.

9. Complementary data

Table S1. Dielectric constants of PTQBT、PTQBT-V、PTQBT-T、PTQBT-2T and PTQT-T at difference frequency for 10^3 (Hz) to 10^6 (Hz).

Polymer	10^3	10^4	10^5	10^6
PTQBT	3.4390	3.4186	3.3367	3.3298
PTQBT-V	3.3946	3.3467	3.2809	3.2508
PTQBT-T	3.6425	3.5714	3.5576	3.5326
PTQBT-2T	3.3467	3.2967	3.2243	3.2029
PTQT-T	3.1903	3.1531	3.1262	3.0873

Table S2. OFET performances and reliability factor of the polymers.

Polymer	Solvent	$T_{annealing}$ [°C]	$\mu_{h,\ max}$ [$\mu_{h,\ avg}$][a)] [cm^2 V^{-1} s^{-1}]	V_{th} [V]	$I_{on/off}$	γ[b)] [%]
PTQBT	CB	N/A	0.042 (0.035 ± 0.048)	–9	10^3-10^4	80
		200	0.12 (0.084±0.021)	–3	10^4-10^5	89
PTQBT-V	CB	N/A	0.036 (0.028 ± 0.0046)	–16	10^4-10^5	77
		200	0.05 (0.042 ± 0.006)	–9	10^3-10^4	80
PTQBT-T	CB	N/A	0.5 (0.45 ± 0.034)	–10	10^4-10^5	97
		200	3.46 (2.93 ± 0.29)	–11	10^3-10^4	82
PTQBT-2T	CB	N/A	0.114 (0.094 ± 0.011)	–10	10^3-10^4	89
		200	0.33 (0.28 ± 0.03)	–10	10^3-10^4	83
PTQBT-T	Anisole	N/A	0.21 (0.176 ± 0.018)	–16	10^3-10^4	81
		150	2.30 (2.01 ± 0.22)	–5	10^3-10^4	80

PTQT-T[c]	DCB	N/A	0.021 (0.018±0.0031)	–8	2×10^5	—[d]
		200	0.54 (0.47 ± 0.074)	–14	5×10^5	—[d]

[a]Average value was based on 10 independent devices and listed in parentheses. [b]Reliability factor. [c]Reported previously. [d]Not available.

Table S3. Summary of green solvents-processed OFET device data by spin-coating deposition method in recent 10 years.

Year	Materials	$\mu_{h,max}$ [cm^{2}V^{-1}s^{-1}]	Solvents	References
2013	P3TEGT	3.5×10^{-5}	Water	*Polym. Chem.* **2013**, 4, 5270
2015	P-DPP-TT(7)SVS(3)	0.5<br>1.03	Ethanol<br>Butanol	*Adv. Funct. Mater.* **2015**, 25, 4844
2017	PPDT2FBT-A	0.01	Ethanol	*Macromolecules* **2017**, 50, 4415
2018	PPDT2FBT-A	0.02	Water	*Adv. Energy Mater.* **2018**, 1802674
2018	DPP-10C$_5$DE	0.05	*o*-Methylanisole	*Macromolecules* **2018**, 51, 4976
2020	P2F2TBT	0.1	Water-Ethanol	*Chem. Mater.* **2020**, 32, 1111
2021	P2b<br>P3b	1.72<br>0.266	*o*-Methylanisole	*ACS Appl. Mater. Interfaces* **2021**, 13, 25175
2022	Fu-F	0.36	anisole	*J. Mater. Chem. C* **2022**, 10, 2616
2024	PTQBT-T	2.30	anisole	This work

10. References

[1] D. Prat, A. Wells, J. Hayler, H. Sneddon, C. R. McElroy, S. Abou-Shehada, P. J. Dunn, *Green Chem.* **2016,** *18*, 288.

[2] D. Prat, O. Pardigon, H.-W. Flemming, S. Letestu, V. Ducandas, P. Isnard, E. Guntrum, T. Senac, S. Ruisseau, P. Cruciani, P. Hosek, *Org. Process Res. Dev.* **2013**, *17*, 1517.

[3] R. K. Henderson, C. Jiménez-González, D. J. C. Constable, S. R. Alston, G. G. A. Inglis, G. Fisher, J. Sherwood, S. P. Binks, A. D. Curzons, *Green Chem.* **2011**, *13*, 854.

[4] Hexemer, A.; Bras, W.; Glossinger, J.; Schaible, E.; Gann, E.; Kirian, R.; MacDowell, A.; Church, M.; Rude, B.; Padmore, H., A SAXS/WAXS/GISAXS Beamline with Multilayer Monochromator. *J. of Phys.: Conf. Ser.* **2010**, *247*, 012007.

[5] Gaussian 09, Revision E.01, Frisch, M. J., Trucks, G. W., Schlegel, H. B., Scuseria, G. E., Robb, M. A., Cheeseman, J. R., Scalmani, G., Barone, V., Mennucci, B., Petersson, G. A., Nakatsuji, H., Caricato, M., Li, X., Hratchian, H. P., Izmaylov, A. F., Bloino, J., Zheng, G., Sonnenberg, J. L., Hada, M., Ehara, M., Toyota, K., Fukuda, R., Hasegawa, J., Ishida, M., Nakajima, T., Honda, Y., Kitao, O., Nakai, H., Vreven, T., Montgomery, J. A., Jr., Peralta, J. E., Ogliaro, F., Bearpark, M., Heyd, J. J., Brothers, E., Kudin, K. N., Staroverov, V. N., Kobayashi, R., Normand, J., Raghavachari, K., Rendell, A., Burant, J. C., Iyengar, S. S., Tomasi, J., Cossi, M., Rega, N., Millam, J. M., Klene, M., Knox, J. E., Cross, J. B., Bakken, V., Adamo, C., Jaramillo, J., Gomperts, R., Stratmann, R. E., Yazyev, O., Austin, A. J., Cammi, R., Pomelli, C., Ochterski, J. W., Martin, R. L., Morokuma, K., Zakrzewski,

V. G., Voth, G. A., Salvador, P., Dannenberg, J. J., Dapprich, S., Daniels, A. D., Farkas, Ö., Foresman, J. B., Ortiz, J. V., Cioslowski, J., Fox, D. J. Gaussian, Inc., Wallingford CT, **2009**.
[6] A. D. Becke, *J. Chem. Phys.* **1992**, *96*, 2155.
[7] a) W. J. Hehre, R. Ditchfield, J. A. Pople, *J. Chem. Phys*. **1972**, *56*, 2257; b) P. C. Hariharan, J. A.Pople, *Theor. Chim. Acta.* **1973**, *28*, 213.
[8] S. Grimme, S. Ehrlich, L. Goerigk, *J. Comput. Chem.* **2011**, *32*, 1456.
[9] G. Kresse, J. Furthmüller, *Phys. Rev. B* **1996**, *54*, 11169.
[10] P. E. Blöchl, *Phys. Rev. B.* **1994**, *50*, 17953.
[11] B. B.-Y. Hsu, C.-M. Cheng, C. Luo, S. N. Patel, C. Zhong, H. Sun, J. Sherman, B. H. Lee, L. Ying, M. Wang, G. Bazan, M. Chabinyc, J.-L. Brédas, A. Heeger, *Adv. Mater.* **2015**, *27*, 7759.
[12] L. Ye, W. Li, X. Guo, M. Zhang, H. Ade, *Chem. Mater.* **2019**, *31*, 6568; b) Z. Wang, Y. Shi, Y. Deng, Y. Han, Y. Geng, *Adv. Funct. Mater.* **2021**, *31*.2104881.
[13] Z. F. Yao, Z. Y. Wang, H. T. Wu, Y. Lu, Q. Y. Li, L. Zou, J. Y. Wang and J. Pei, *Angew. Chem. Int. Ed.*, **2020**, *59*, 17467.
